# *FvatfA* regulates growth, stress tolerance as well as mycotoxin and pigment productions in *Fusarium verticillioides*

**DOI:** 10.1007/s00253-020-10717-6

**Published:** 2020-07-27

**Authors:** Zsuzsa Szabó, Klaudia Pákozdi, Katalin Murvai, Tünde Pusztahelyi, Ádám Kecskeméti, Attila Gáspár, Antonio F. Logrieco, Tamás Emri, Attila L. Ádám, Éva Leiter, László Hornok, István Pócsi

**Affiliations:** 1 Department of Molecular Biotechnology and Microbiology, Institute of Biotechnology, Faculty of Science and Technology, University of Debrecen, Debrecen, Hungary; 2 Doctoral School of Biological Sciences, Faculty of Agricultural and Environmental Sciences, Szent István University, Gödöllő, Hungary; 3 Doctoral School of Nutrition and Food Sciences, Faculty of Medicine, University of Debrecen, Debrecen, Hungary; 4 Central Laboratory of Agricultural and Food Products, Faculty of Agricultural and Food Sciences and Environmental Management, University of Debrecen, Debrecen, Hungary; 5 Department of Inorganic and Analytical Chemistry, Faculty of Science and Technology, University of Debrecen, Debrecen, Hungary; 6 Institute of Sciences of Food Production (ISPA-CNR), Bari, Italy; 7 Plant Protection Institute, Centre for Agricultural Research, Budapest, Hungary; 8 Faculty of Agricultural and Environmental Sciences, Szent István University, Gödöllő, Hungary

**Keywords:** Vegetative growth, Invasive growth, Conidiogenesis, Oxidative stress, Cell wall integrity stress, Mycotoxin production, Pigment production

## Abstract

*FvatfA* from the maize pathogen *Fusarium verticillioides* putatively encodes the *Aspergillus nidulans* AtfA and *Schizasaccharomyces pombe* Atf1 orthologous bZIP-type transcription factor, FvAtfA. In this study, a *ΔFvatfA* deletion mutant was constructed and then genetically complemented with the fully functional *FvatfA* gene. Comparing phenotypic features of the wild-type parental, the deletion mutant and the restored strains shed light on the versatile regulatory functions played by FvAtfA in (i) the maintenance of vegetative growth on Czapek-Dox and Potato Dextrose agars and invasive growth on unwounded tomato fruits, (ii) the preservation of conidiospore yield and size, (iii) the orchestration of oxidative (H_2_O_2_, menadione sodium bisulphite) and cell wall integrity (Congo Red) stress defences and (iv) the regulation of mycotoxin (fumonisins) and pigment (bikaverin, carotenoid) productions. Expression of selected biosynthetic genes both in the fumonisin (*fum1*, *fum8*) and the carotenoid (*carRA*, *carB*) pathways were down-regulated in the *ΔFvatfA* strain resulting in defected fumonisin production and considerably decreased carotenoid yields. The expression of *bik1*, encoding the polyketide synthase needed in bikaverin biosynthesis, was not up-regulated by the deletion of *FvatfA* meanwhile the *ΔFvatfA* strain produced approximately ten times more bikaverin than the wild-type or the genetically complemented strains. The abolishment of fumonisin production of the *ΔFvatfA* strain may lead to the development of new-type, biology-based mycotoxin control strategies. The novel information gained on the regulation of pigment production by this fungus can be interesting for experts working on new, *Fusarium*-based biomass and pigment production technologies.

**Table:** 

**Key points** *• FvatfA regulates vegetative and invasive growths of F. verticillioides.* *• FvatfA also orchestrates oxidative and cell wall integrity stress defenses.* *• The ΔFvatfA mutant was deficient in fumonisin production.* *• FvatfA deletion resulted in decreased carotenoid and increased bikaverin yields.*

**Electronic supplementary material:**

The online version of this article (10.1007/s00253-020-10717-6) contains supplementary material, which is available to authorized users.

## Introduction

The human bZIP-type activating transcription factor ATF-2, which binds to the cAMP-responsive promoter sequences, plays a complex role in the adaptation of various cell types to environmental stress, stress-induced epigenome changes, and also in oncogenesis (Vlahopoulos et al. 2008; Seong et al. 2012; Krifka et al. 2013). ATF-2 activity is positively regulated by the stress-activated protein kinases p38 and JNK (Vlahopoulos et al. 2008; Seong et al. 2012; Krifka et al. 2013). Homologs of human ATF-2 can even be found in evolutionarily distant eukaryotes, including fungi. In the fission yeast, *Schizosaccharomyces pombe*, the ATF-2 homolog Atf1 is activated by the Sty1 mitogen-activated protein kinase (MAPK), an ortholog of the *Saccharomyces cerevisiae* Hog1 MAPK, in response to a wide spectrum of environmental stress and is the master regulator of fission yeast’s Environmental Stress Response (Chen et al. 2003; Gasch 2007). Atf1 and its heterodimeric bZIP transcription factor partner, Pcr1 orchestrate osmotic, oxidative, heat shock and nitrogen deprivation stress responses through binding to promoters of a wide array of stress response genes and also via modulating chromatin architecture (Sansó et al. 2008, 2011). Atf1 is indispensable in the entry of fission yeast into the stationary growth phase and also in sexual development (Takeda et al. 1995; Shiozaki and Russel 1996).

In the saprophytic filamentous fungus model organism, *Aspergillus nidulans*, the AtfA transcription factor, a true ortholog of the fission yeast’s Atf1 (Balázs et al. 2010) interacts with the SakA/HogA MAPK and regulates various stress responses. Furthermore, it also affects asexual development and contributes to the maintenance of conidial viability and the stress tolerance of both conidia and vegetative tissues (Hagiwara et al. 2008, 2009; Balázs et al. 2010; Lara-Rojas et al. 2011). AtfA is a key regulator of conidial dormancy and stress tolerance in various other *Aspergillus* spp. as well (Sakamoto et al. 2009; Hagiwara et al. 2014, 2016). AtfA has a stress-specific impact on the expression of a number of gene groups with versatile physiological functions, e.g. through the modulation of stress signal transduction (Emri et al. 2015; Orosz et al. 2017; Antal et al. 2019). AtfA is a key player in the regulation of oxidative stress response in menadione-exposed *A. nidulans* cultures, when fungal cells combat the deleterious effects of increasing intracellular superoxide anion radical concentrations (Pócsi et al. 2005; Pusztahelyi et al. 2011; Emri et al. 2015; Orosz et al. 2017; Antal et al. 2019). It is noteworthy that some secondary metabolite gene clusters were stress inducible (e.g. asperfuranone, terriquinone) or stress repressible (e.g. austinol) in *A. nidulans* and the deletion of *atfA* made some other clusters stress responsible as well (either stress repressible or inducible) (Emri et al. 2015; Antal et al. 2019). Importantly, many primary metabolic pathways (e.g. amino acid and fatty acid metabolic processes, tricarboxylic acid cycle) were also influenced by AtfA under various types of oxidative stress in *A. nidulans* (Orosz et al. 2017).

In plant pathogenic fungi, Atf1/AtfA orthologous transcription factors have also been shown to have a role in (i) the maintenance of vegetative growth (*Magnaporthe oryzae* Moatf1 (Guo et al. 2010); *Fusarium graminearum* FgAtf1 (Nguyen et al. 2013; Jiang et al. 2015)), (ii) regulation of sexual (*F. graminearum* FgAtf1 (Nguyen et al. 2013; Jiang et al. 2015)) and asexual (*Botrytis cinerea* BcAtf1 (Temme et al. 2012), *F. graminearum* FgAtf1 (Jiang et al. 2015)) developments, (iii) environmental (oxidative, osmotic, cell wall integrity) stress defence(*Claviceps purpurea* CPTF1 (Nathues et al. 2004), *B. cinerea* BcAtf1 (Temme et al. 2012), *F. graminearum* FgAtf1 (Nguyen et al. 2013; Jiang et al. 2015), *Fusarium oxysporum* Foatf1 (Qi et al. 2013)), (iv) virulence and the modulation of plant defence(*C. purpurea* CPTF1 (Nathues et al. 2004), *M. oryzae* Moatf1 (Guo et al. 2010), *B. cinerea* BcAtf1 (Temme et al. 2012), *F. graminearum* FgAtf1 (Nguyen et al. 2013; Jiang et al. 2015), *F. oxysporum* Foatf1 (Qi et al. 2013)), (v) release of enzymes like laccases, peroxidases and catalases (*C. purpurea* CPTF1 (Nathues et al. 2004), *M. oryzae* Moatf1 (Guo et al. 2010), *F. oxysporum* Foatf1 (Qi et al. 2013)) as well as (vi) control of primary (*B. cinerea* BcAtf1 (Temme et al. 2012)) and secondary (*B. cinerea* BcAtf1 (Temme et al. 2012), *F. graminearum* FgAtf1 (Nguyen et al. 2013; Jiang et al. 2015)) metabolism.

The transcriptional regulation of secondary metabolite gene clusters by Atf1/AtfA orthologs may be either positive or negative depending on species and culture conditions (Emri et al. 2015; Antal et al. 2019). Deoxynivalenol production increased in toxin induction in vitro cultures but decreased in wheat heads infected with a *F. graminearum ΔFgatf1* gene deletion mutant in comparison with the wild-type parental strain (Nguyen et al. 2013; Jiang et al. 2015). The *ΔFgatf1* strain also produced less zearalenone *in planta*, whereas in artificial cultures, the mutant and the wild-type strain produced similar amounts of this mycotoxin (Nguyen et al. 2013). Interestingly, the *ΔFgatf1* strain also overproduced the golden yellow pigment, aurofusarin on agar plates (Nguyen et al. 2013). Expression of selected members of the deoxynivalenol, zearalenone and aurofusarin gene clusters paralleled with alterations in mycotoxin and pigment productions both in *in vitro* and *in vivo* studies (Nguyen et al. 2013). Nevertheless, while deletion of *B. cinerea bcatf1* resulted in a significant overproduction of botrydial, botryendial and botcinin A, potent phytotoxins of the fungus in axenic cultures, no physical interactions could be demonstrated between BcAtf1 and the promoters of selected phytotoxin biosynthetic genes by yeast one-hybrid analyses (Temme et al. 2012). This means that both direct and indirect regulatory effects of the Atf1/AtfA-type transcription factors may have an impact on secondary metabolite production in plant pathogenic fungi.

*Fusarium verticillioides* is a common pathogen of maize causing seedling blight, stalk and ear rot and produces a group of harmful polyketide-type mycotoxins called fumonisins (Picot et al. 2010; Woloshuk and Shim 2013; Blacutt et al. 2018). Because fumonisins interfere with the biosynthesis of sphingolipids (Liu et al. 2019), there are many organs affected by them including the liver, kidneys as well as the respiratory and nervous systems in both humans and animals (Wu et al. 2014; Nair 2017; Kouzi et al. 2018; Ponce-García et al. 2018). Fumonisins may also cause oesophageal (Kamangar et al. 2009; Kigen et al. 2017) and renal (Müller et al. 2012; Wu et al. 2014; Barnett and Cummings 2018) cancers as well as neural tube defects (Wu et al. 2014; Ponce-García et al. 2018; Lumsangkul et al. 2019). A deeper understanding of the molecular regulation of fumonisin biosynthesis in toxigenic fusaria (Butchko et al. 2012; Lazzaro et al. 2012; Woloshuk and Shim 2013; Gu et al. 2017; Gil-Serna et al. 2019) may lead to the development of atoxigenic strains with possible biocontrol potential or to the utilization of RNA interference technologies to silence mycotoxin biosynthesis genes (Alberts et al. 2016).

*F. verticillioides* also produces various pigments like the red-coloured secondary metabolite bikaverin (Chelkowski et al. 1992; Choi et al. 2008; Butchko et al. 2012; Lazzaro et al. 2012) with antimicrobial (Balan et al. 1970; Son et al. 2008; Deshmukh et al. 2014; Sondergaard et al. 2016; Lebeau et al. 2019) and anticancer (Fuska et al. 1975; Zhan et al. 2007; Limón et al. 2010) activities and also light-inducible carotenoids (Ádám et al. 2011) with potential biotechnological significance (Gmoser et al. 2017). Atoxigenic *Fusarium* strains are considered possible industrial pigment producers or even they produce mycoproteins for human consumption fortified with bioactive carotenoids (Gmoser et al. 2017).

In this paper, we report on the deletion of the *FvatfA* gene in *F. verticillioides* and the effects of this gene deletion on growth, invasive growth, asexual sporulation, abiotic stress tolerance as well as fumonisin, carotenoid and bikaverin production of this maize pathogen fungus. The potential biocontrol and industrial significance of the complete loss of fumonisin production paralleled with bikaverin overproduction as observed in the *ΔFvatfA* mutant is also discussed.

## Materials and methods

### Fungal strains, culture media and growth conditions

Conidiospore suspensions of *F. verticillioides* wild-type strain, FGSC 7600 its deletion mutant, *ΔFvatfA* and two ectopic-complemented strains generated in this study were stored in 50% glycerol at − 70 °C. To prepare starter inocula for growth assays, stress sensitivity studies, RNA isolation and secondary metabolite analyses, fungi were grown on Czapek-Dox agar medium (containing 20 g L^−1^ sucrose) for 7 days at 25 °C. Conidiospores were scraped in sterile water containing 9 g L^−1^ NaCl and 100 μL L^−1^ TWEEN-80, passed through two layers of Miracloth (Merck-Millipore) and then quantified using a haemocytometer. For genomic DNA isolation, fungi were grown in YPG medium (10 g L^−1^ peptone, 3 g L^−1^ yeast extract, 20 g L^−1^ glucose) for 3 days at 28 °C with shaking at 3.3 Hz (200 rpm) frequency.

### Invasive growth on tomato fruits

Invasive growth of the fungi was assessed by placing mycelial blocks (6 mm in diameter) cut out from 4-day-old fungal cultures grown on Czapek-Dox agar plates onto surface sterilized tomato fruits (four replicates per strain) and incubated at room temperature (Di Pietro et al. 2001). Colony diameters were measured and photographs were taken at 72 and 96 h post inoculation (hpi). All experiments were repeated three times.

### Multiple sequence alignment and phylogenetic tree construction

The full sequence of FvAtfA was downloaded from the National Center for Biotechnology Information (NCBI) *F. verticillioides* FGSC 7600 online database. Orthologous sequences (listed in Supplementary Table S1) from different organisms were also obtained from NCBI by BLAST homology search using the standard blastp algorithm (McGinnis and Madden 2004). The amino acid sequences were aligned with the MEGA MUSCLE algorithm (Kumar et al. 2018). Gap open penalties were set to − 10. The aligned protein sequences were trimmed with TrimAI. The evolutionary history was inferred by using the Maximum Likelihood method and JTT matrix-based model (Jones et al. 1992). The molecular mass and isoelectric point of the FvAtfA protein was calculated using the Isoelectric Point Calculator (Kozlowski 2016; http://isoelectric.org/calculate.php). bZIP domains of the protein sequences (according to uniprot.org domain analysis with InterPro annotation) were aligned using the NCBI Constraint-based Multiple Alignment Tool, and amino acids with similar properties were visualized with RasMol Amino Acid Colors (Ray 2005).

### Generation of *FvatfA* disruption mutant and complemented strains

The deletion construct was generated by the double-joint PCR method (Yu et al. 2004) to replace the *FvatfA* ORF in *F. verticillioides* strain FGSC 7600. First, 1524 bp 5’- and 1557 bp 3’-flanking regions of the *FvatfA* gene were amplified from *F. verticillioides* genomic DNA using Expand Long Polymerase. The primers FvatfAupfwd and FvatfAupkimrev were used to amplify the 5’-flanking region, and primers FvatfAdownkimfwd and FvatfAdownrev were employed to amplify the 3’-flanking region. Primers used in this study are listed in Table S2. Simultaneously, the hygromycin B phosphotransferase-encoding gene (*hph*, *Escherichia coli*) was amplified from plasmid vectors pBP15 (Sagaram et al. 2007), respectively, using the primers M13F and M13R (Supplementary Table S2). Subsequently, the three amplicons were mixed together in a single tube in 1:3:1 (5’-fragment:marker:3’-fragment) molar ratio and joined by PCR without any primers. Finally, nested primers FvatfAnestedfwd and FvatfAnestedrev were used to amplify the 4.1-kb amplicon carrying the *hph* marker fused to the *FvatfA* flanking regions. This fused product was used as *FvatfA* disruption construct. The polyethylene glycol (PEG)-mediated protoplast transformation method was used to transform the wild-type strain as previously described by Sagaram et al. (2007). Transformants were regenerated in 8 mL regeneration medium (343 g L^−1^ sucrose, 0.2 g L^−1^ yeast extract) overnight at 28 °C at 60 rpm. Regenerated colonies were selected on regeneration agar (343 g L^−1^ sucrose, 0.2 g L^−1^ yeast extract, 10 g/L agar) containing 100 μg/mL hygromycin. To confirm genetic homogeneity, all the transformants were regrown from a single conidium on Czapek-Dox agar plates containing 100 μg mL^−1^ hygromycin. A 3-mm Ø agar plug was taken from each transformant and placed into glass test tubes with caps containing 2 mL of YPG medium. Tubes were incubated in a rotary shaker overnight at 28 °C at 200 rpm. Genomic DNA was isolated from mycelial mat collected by centrifugation. Emerald PCR was carried out using the FvatfAupfwd and FvatfAdownrev primers (Supplementary Table S2). PCR products were digested with *Eco*RV and *Nde*I. Strains carrying the deletion cassettes were stored as a glycerol stock at − 70 °C. Southern blot analysis was also performed to confirm single-copy integration of the deletion cassette at the homologous recombinant site of the *FvatfA* gene.

The *FvatfA* deletion strain *ΔFvatfA* (*ΔFvatfA :: hph*) was complemented with a wild-type *FvatfA* gene fused to the geneticin (G418)-resistance gene (*gen*, *E. coli*) amplified with primers M13F and M13R from pBS-G; whereas *FvatfA* was amplified from genomic DNA with FvatfAcompkimfwd and FvatfAcomprev (Supplementary Table S2) using Expand Long Polymerase. These two amplicons were fused by a single-joint PCR strategy to generate the complementation construct (Shim et al. 2006; Yu et al. 2004). The joined-PCR product was amplified with primers M13F and FvatfAcomprev and used for transformation to complement *ΔFvatfA.* After genomic DNA isolation, Emerald PCR was performed using M13F–M13R and FvatfAcompkimfwd–FvatfAnestedrev to amplify the geneticin cassette and the *FvatfA* gene with UTR regions, respectively. PCR product of FvatfAcompkimfwd–FvatfAnestedrev was digested with *Eco*RI, *Xho*I and *Ssp*I. Single copy integration of the complementation cassette was confirmed by qPCR.

### Nucleic acid manipulations and Southern blot assay

Plasmids pBP15 and pBS-G containing the marker genes were kindly provided by Professor Won-Bo Shim (Texas A&M University). Plasmid DNA was isolated from *E. coli* grown in 2 mL of LB medium for 18 h at 37 °C using the NucleoSpin Plasmid Kit (Macherey-Nagel). Fungal genomic DNA was extracted following the protocol of Leslie and Summerell (2006). All PCR assays were performed in a Biometra Thermal Cycler (T Professional ThermoCycler) using Expand Long Polymerase (Roche) or EmeraldAmp MAX PCR Master Mix (Takara).

In Southern blot assays, genomic DNA digested with *Sca*I and separated by electrophoresis was transferred onto Immobilon-NY^+^ membrane (Millipore) and probed with a DIG-labelled DNA fragment amplified from the genomic DNA using FvatfAupfwd and FvatfAupkimrev primers (Supplementary Table S2). Southern analysis was performed using the DIG DNA Labeling and Detection Kit (Roche).

### Copy-number determination

Copy number of *FvatfA* in the complemented strains was determined by quantitative real-time PCR assay according to Herrera et al. (2009) with minor modifications, using *FvmnSOD* (putatively coding for manganese superoxide dismutase) as a single-copy reference gene. Total DNA was extracted from lyophilised fungal mycelia. DNA was quantified using a NanoDrop spectrophotometer (Thermo Fisher Scientific, Waltham, MA). Five serial 1:2 dilutions (320, 160, 80, 40 and 20 ng 7 μL^−1^) of DNA from the complemented strains were used to generate standard curves of *C*_T_ (threshold cycle) value against the log DNA concentration in each well.

qPCRs were performed in triplicates using a LightCycler 480 (Roche). Quantitative RT-PCR was performed in a total volume of 13 μL, composed of 10 μL Fast SYBR Green master mix (Applied Biosystems by Life Technologies), 0.4 μL reverse primer, 0.4 μL forward primer (the primers are listed in Supplementary Table S2) and 2.2 μL nuclease-free water in each well of the 96-well plate. PCR cycles were performed according to the following protocol: 1. 95 °C for 2 min; 40× cycles, 95 °C for 5 s, 51 °C for 10 s, 65 °C for 20 s; 95 °C for 15 s, 51 °C for 15 s, 95 °C continuous and 37 °C for 1 s.

Equation (1) taken from the Eq. (2) for a line was constructed by plotting the standard curve of the log quantity versus its corresponding C_*T*_ value

1 $$ {C}_T\kern0.5em =\kern0.5em m\kern0.2em \left(\log \kern0.2em \mathrm{quantity}\right)\kern0.5em +\kern0.5em b $$

2 $$ y\kern0.5em =\kern0.5em mx\kern0.5em +\kern0.5em b $$

If the curve demonstrated an *r*^2^ value of > 0.980, the standard curve was then used to determine the sensitivity, primer efficiencies, the dynamic range as well as the specificity and reproducibility of each assay. The copy numbers of the *FvatfA* gene were determined by the absolute quantitation method, by which total copies were first calculated using Eq. (3).

3 $$ FvatfA\ \mathrm{copies}={10}^{\left(\left[{\mathrm{C}}_T-\mathrm{b}\right]/\mathrm{m}\right)} $$

The number of *FvatfA* copies per genome was then determined by Eq. (4):

4 $$ FvatfA\ \mathrm{copies}\ \mathrm{per}\ \mathrm{genome}=\left(\mathrm{total}\ \mathrm{copies}\ \mathrm{of}\  FvatfA\right)/\left(\mathrm{total}\ \mathrm{copies}\ \mathrm{of}\  FvmnSOD\right) $$

### Comparison of phenotypes

To determine growth rate, Czapek-Dox agar or PDA plates were inoculated with conidial suspensions (1 × 10^5^ conidia in 5 μL) of the fungi. Plates were incubated in the dark for 6 days at 25 °C. Colony diameters were measured, and the results of triplicated assays were analysed.

Colonies were scraped with sterile distilled water and filtered through two layers of Miracloth. Microscopic pictures (× 400) were then taken using Thoma cell counter. The arc lengths and diameters of 100–100 spores in three biological replicates (300 in total) were measured using the ImageJ software. Mean values calculated in the replicate experiments were used in further statistical analysis.

To quantify conidiation, agar cylinders were excised with a cork borer (8 mm diameter) from Czapek-Dox agar plates, vortexed in Eppendorf tube with 1 mL sterile water, and conidia were counted with a haemocytometer (Shim et al. 2006). Microphotographs were taken with an Olympus BX51 microscope equipped with a DP70 digital camera.

### Spore viability assay

Spore suspensions (1 × 10^3^ mL^-1^) were incubated at 25, 42 and 45 °C for 60 min or at 4 °C for 7 days; 100 μL suspensions from each tube were plated on Czapek-Dox agar, and colonies were counted after 2 days of incubation at 25 °C (Choi and Xu 2010).

### Stress sensitivity tests

To estimate stress sensitivities 1 × 10^5^ conidia harvested from 7-day-old cultures were point inoculated on Czapek-Dox agar plates, supplemented with one of the following stress-generating agents (Nagygyörgy et al. 2014; Leiter et al. 2016; Orosz et al. 2018): sorbitol (a nonionic osmolyte; 0.1–2 M), NaCl (an ionic osmolyte; 0.1–1.5 M), KCl (an ionic osmolyte; 0.1–1.5 M), CdCl_2_ (elicits heavy metal stress; 0.1–0.4 mM), menadione sodium bisulphite (MSB, causes superoxide stress; 0.2–1.4 mM), diamide (triggers glutathione-glutathione disulphide redox imbalance; 0.1–1 mM), *tert*-butyl hydroperoxide (initiates lipid peroxidation; *t*BOOH, 0.2–0.8 mM), H_2_O_2_ (causes peroxide stress; 10–50 mM) and Congo Red (generates cell wall integrity stress; 5–25 μM) at various concentrations as indicated in parentheses. Colony diameters were measured after 6 days of incubation at 25 °C, and relative growth was calculated as percentage of growth of the wild-type strain. Growth inhibition recorded for the mutant was always compared with that of the FGSC 7600 wild-type strain.

### Fumonisin analysis

Five milliliters of Myro medium (Han et al. 2014) in 6-well plates (Corning) were inoculated with 50 μL aliquots of spore suspensions containing 5 × 10^6^ spores. Cultures were incubated in the dark at 25 °C for 14 days in static conditions. Culture supernatants were collected by centrifugation at 3000 × *g* at 4 °C for 10 min. The pelleted fungal biomass was lyophilized and weighed.

Fumonisin B_1_ (FB1) and fumonisin B_2_ (FB2) concentrations of the supernatants were measured by capillary electrophoresis (7100 CE System, Agilent, Waldbronn, Germany) coupled to an electrospray mass spectrometer (maXis II UHR ESI-QTOF MS instrument, Bruker, Karlsruhe, Germany) operated by OpenLAB CDS Chemstation software (Agilent). Hyphenation was performed with a CE-ESI Sprayer interface (G1607B, Agilent). Sheath liquid was transferred with a 1260 Infinity II isocratic pump (Agilent). CE instrument was operated by OpenLAB CDS Chemstation software. The following parameters were used for CE-MS analysis, 90 cm × 50 μm i.d. fused silica capillary; BGE: 40 mM HCOONH_4_/NH_3_ (pH = 9.5); SL: iPrOH:water = 1:1 with 0.1% formic acid; sheath liquid flow rate, 10 μL min^−1^; voltage, 20 kV; injection, 50 mbar × 10 s. The MS method was tuned according to the desired mass range (best sensitivity between 650 and 800 m/z for fumonisin B1 and B2, complete scan was between 400 and 1200 m/z). MS conditions, positive mode; nebulizer pressure, 0.5 bar; dry gas temperature, 200 °C; dry gas flow rate, 4 L min^−1^; capillary voltage, 4500 V; end plate offset, 500 V; and spectra rate, 3 Hz. Mass spectra were recorded by otofControl version 4.1 (build: 3.5, Bruker) and processed by Compass DataAnalysis version 4.4 (build: 200.55.2969).

Electropherograms were extracted at the masses of the examined analytes (722.3950 and 706.4000 ± 0.005 m/z, for FB1 and FB2, respectively). Peaks on the extracted ion electropherograms were integrated automatically, without further background correction. The linear calibration curve was plotted based on intensities obtained for standard FB1 and FB2.

### Determination of carotenoids

Light is the major regulator of carotenoid biosynthesis, but low N/C ratio has also a positive effect on carotenogenesis (Avalos et al. 2017) and, therefore we used DG minimal medium as previously described (Hornero-Mendez et al. 2018). Fungi were cultured in 100 mL liquid DG minimal medium, and the cultures were kept in Erlenmeyer flasks thermostatically maintained at 25 °C, illuminated with cool white fluorescent light (80 μmol photons m^−2^ s^−1^) on a rotary shaker (2.5 Hz shaking frequency). Biomass of the cultures was separated by filtration of 5 mL culture on Whatman paper No. 5 and used for dry cell mass determination. Mycelia were collected on Miracloth into 15 mL Falcon tubes and then freeze-dried; 100 mg dried mycelium was disrupted in a BeadBeater homogenizer (Biospecs) at 66.7 Hz beating frequency, 30 s with 100 mg quartz sand and 1 mL acetone. The homogenized samples were centrifuged at 16,000×*g* for 5 min,repeatedly, until bleaching of the samples. The collected supernatants were evaporated in Rotavapor (Büchi) at 40 °C, and the dried extracts were dissolved in 2 mL petroleum ether, loaded onto anhydrous Al_2_O_3_ column (1 × 1 cm) and eluted by 10 mL petroleum ether. Absorbance was measured at 450 nm. Carotenoid content was calculated using a correlation shown below in Eq. (5):

5 $$ \mathrm{Carotenoids}\left(\upmu \mathrm{g}/\mathrm{g}\right)=\frac{A\times {V}_1\times {V}_2\times {10}^4}{A_{1\ \mathrm{cm}}^{1\%}\times {V}_3\times m} $$

where *A* is the absorbance; *V*_1_ is the volume of the eluate; *V*_2_ is the total volume of the extract; *V*_3_ is the volume of the aliquot loaded onto the column; ${A}_{1\ \mathrm{cm}}^{1\%}$ = 2592 (β-carotene extinction coefficient in petroleum ether); and *m* is the mass of the sample.

### Bikaverin measurement

Bikaverin production is stimulated by low N/C ratio in the medium; the presence of calcium and sucrose also positively affects the biosynthesis this polyketide pigment (Limón et al. 2010). For bikaverin measurement we followed the method described by Bell et al. (2003). Briefly, 100 mL liquid medium (basal medium for toxin production containing 20 g L^−1^ sucrose and 140 mg L^−1^ urea as carbon and nitrogen sources, respectively, and also supplemented with 500 mg L^−1^ CaCO_3_, pH 5.0; Bell et al. (2003)) was inoculated with 1 × 10^8^ conidia scraped from 7-day-old colonies and grown as shaken culture (3.33 Hz) for 5, 7 and 9 days at 28 °C. Biomass of the cultures was separated by filtration of 5 mL culture on Whatman paper No. 5 and used for dry mass determinations. Bikaverin concentration was determined by diluting the supernatants (1 mL) with 3 mL extraction solvent (acetone + 1 M sulfuric acid 90:10); absorbance of the samples was measured at 500 nm spectrophotometrically. Calibration curve was prepared in the range of 0.488–125 μg/mL using purified bikaverin (Sigma) as standard.

### Measurement of gene expression

Expression levels of genes involved in fumonisin, bikaverin and carotenoid biosynthetic pathways were measured by RT-PCR. Mycelial samples were collected after incubation for 14 days in Myro medium (for the analysis of *fum1*, *fum8* and *fum21* genes), 3 and 5 days in bikaverin-inducing medium (for *bik1* gene analysis) and 4 days of incubation in the dark in DG medium followed with 2 h illumination (for the analysis of *carRA*, *carB* and *carT* genes) and stored at − 80 °C. (For further information on the function of the genes assayed in gene expression experiments, consult Supplementary Table S3). After lyophilisation, RNA samples were isolated with TRI reagent (Invitrogen) (Chomczynski 1993). Real-time polymerase chain reaction with the Xceed qPCR SG 1-step Kit (IAB) was carried out using the LightCycler 480 Real-Time PCR System (Roche) according to the manufacturer’s recommendations with 500 ng of total RNA per reaction in 40 cycles. The steps for the qRT-PCR reaction were as follows: (1) reverse transcription, 45 °C for 10 min; (2) PCR initial activation step, 95 °C for 2 min; (3) DNA denaturation, 95 °C for 5 s; (4) annealing, 51 °C for 10 s; and (4) extension, 65 °C for 30 s and 40 cycles (primer list in this study see Supplementary Table S2). In each RNA sample, *tef1* (FVEG_02381) transcripts were also quantified as reference gene transcripts (Supplementary Table S2). Relative transcript levels were calculated by the ‘delta method’ where ΔC_*T*_ is the *C*_T_ reference gene − *C*_T_ gene of interest and *C*_T_ stands for the qRT-PCR cycle numbers corresponding to the crossing points. For statistical analysis, the mean ± SD values were calculated from three independent experiments. Relative transcript levels were examined using the following other reference genes as well: *tub2* (FVEG_04081) and *cyp2* (FVEG_00403) with similar results.

**Table 1:** *FvatfA* gene copy-number determination in the complemented *F. verticillioides FvatfA* ‘C (H7) and (H9) strains

Strains	*FvatfA* (FVEG_02866)^a^	R^2^	*FvmnSOD* (FVEG_11192)^a^	R^2^	Copy number
*FvatfA* ‘C (H7)	y = − 3.31*x* + 26.74	0.99	y = − 2.93*x* + 26.62	0.98	1.00 ± 0.02
*FvatfA* ‘C (H9)	y = − 3.49*x* + 26.17	0.97	y = − 3.32*x* + 26.33	0.98	1.00 ± 0.01

In these gene copy-number determinations, the gene FVEG_11192 putatively encoding the manganese superoxide dismutase of *F. verticillioides* (FvMnSOD) was used as a single copy reference gene. The number of FVEG_02866 (*FvatfA*) per genome was determined by the equation FVEG _ 02866 (*FvatfA*) per genome = (total copies of FVEG _ 02866 (*FvatfA*)}/{total copies of FVEG _ 11192 (*FvmnSOD*)}

^a^The equations C_*T*_ = m (log quantity) + b were constructed by plotting the standard curve of log quantity versus its corresponding C_*T*_ value, where *y* is the C_*T*_ value, m is the slope, x is the log(quantity) and b is the intercept

### Promoter analysis

The genes listed in Supplementary Table S4 were obtained with 2 kb 5’-upstream sequence from the NCBI *F. verticillioides* online database. Prediction of genes was carried out using the FGENESH pipeline with generic *Fusarium*-specific gene-finding parameters (http://linux1.softberry.com/berry.phtml?topic=fgenesh&group=programs&subgroup=gfind) (Solovyev et al. 2006). In this analysis, transcription start site (TSS), polyadenylation site (PolA) and the position of translation start (ATG) were also predicted for each gene. ATF/CREB family transcription factors can recognize DNA containing the cAMP-responsive element (CRE) consensus sequence TGACGTCA (Loeken 1993; Kvietikova et al. 1995; Sakamoto et al. 2008; Hong et al. 2013). Standard promoter regions were defined as the − 1000/+ 50-bp sequences around TSS or between the 5’-end of the up-stream intergenic region (if this region was < 1000 bp) and + 50 bp down-stream of TSS (Wolf et al. 2016).

Putative transcription factor binding sites were identified in the promoter regions and also in the 5’-untranslated region (between the transcription and translation start sequences; Roze et al. 2011; Hong et al. 2013). ATF/CREB promoter motifs were searched using the PROMO version 3.0.2 online tool (Messeguer et al. 2002; Farré et al. 2003) by constructing specific binding site weight matrices from TRANSFAC 8.3 database (http://alggen.lsi.upc.es/cgi-bin/promo_v3/promo/promoinit.cgi?dirDB=TF_8.3). Maximum matrix dissimilarity rate in PROMO was set to 15%, and the factor’s and site’s species were set to ‘all factors’ and ‘all sites’, respectively.

### Statistical analysis

Unless otherwise indicated, all experiments were carried out with three biological replicates, and mean ± SD values are presented.

Statistical differences between the strains were tested by one-way ANOVA followed by Tukey post hoc test using the ‘ANOVA’ and ‘Tukey HSD’ functions of R project (http://www.R-project.org/) in the case of the following features: ‘vegetative growth’, ‘spore production’, ‘spore viability’, ‘spore size’, ‘secondary metabolite production’ and ‘gene expression’.

The results of the abiotic stress tolerance were analysed by both one-way ANOVA (followed by Tukey post hoc test) applied on relative growth data (expressed as percentage of growth of the wild-type strain in the same experiment) or two-way ANOVA (also followed by Tukey post hoc test) applied on absolute growth data (colony diameters) recorded in untreated and treated cultures.

Overall differences among strains were analysed by principal component analysis (PCA) using the ‘prcomp’ function of R project. In these calculations, mean values presented in Supplementary Table S5 were used.

## Results

### Characterization of the *FvatfA* gene

A homology search using the NCBI BLASTp algorithm was carried out based on the *A. nidulans* AtfA (Locus ID: AN2911) protein sequence to identify the gene encoding FvAtfA, the AtfA orthologous protein of *F. verticillioides*. The identified FvAtfA protein (encoded by the FVEG_02866 locus in the *F. verticillioides* FGSC 7600 genome; *E* value 3*e*−96) has a predicted molecular mass of 41 kDa and an isoelectric point of 8.7. FvAtfA contains a common bZIP domain typically present in the *Homo sapiens* ATF-2, *S. pombe* Atf1 and *A. nidulans* AtfA orthologous transcription factors (Supplementary Fig. S1; Supplementary Table S1). As expected, *F. verticillioides* FvAtfA shared nearly the same position on the phylogenetic tree with *F. oxysporum* FoAtf1 and *F. graminearum* FgAtf1 (Supplementary Fig. S1).

### Generation of *ΔFvatfA* gene deletion mutant and *FvatfA* ‘C-complemented strains

To elucidate the physiological functions of *FvatfA,* we deleted the gene using the double-joint PCR method of Yu et al. (2004). The *F. verticillioides ΔFvatfA* deletion strain (*ΔFvatfA::hph*) was generated by replacing the *FvatfA* ORF with the hygromycin phosphotransferase gene of *E. coli* as a selective marker (Supplementary Fig. S2). After transforming protoplasts with the gene deletion cassette, hygromycin-resistant colonies were selected and screened for *FvatfA* gene deletion by PCR. Homologous recombination was verified by Southern analysis to demonstrate the proper deletion of the *FvatfA* ORF region and the single-copy integration of the deletion cassette (Supplementary Fig. S2). *FvatfA-*complemented strains were generated by reintroducing the *FvatfA* ORF sequence (with its promoter and terminator regions) together with geneticin phosphotransferase (*gen*, from transposon Tn5) as a marker gene into the *ΔFvatfA* strain. From the successfully complemented *FvatfA* ‘C strains, H7 and H9 were selected for physiological studies. The copy number of *FvatfA* was determined both in H7 and H9 using the qRT-PCR protocol of Herrera et al. (2009); these experiments confirmed the single-copy integration of *FvatfA* (Table 1).

### *FvatfA* deletion leads to subnormal vegetative and invasive growth

**Fig. 1:**
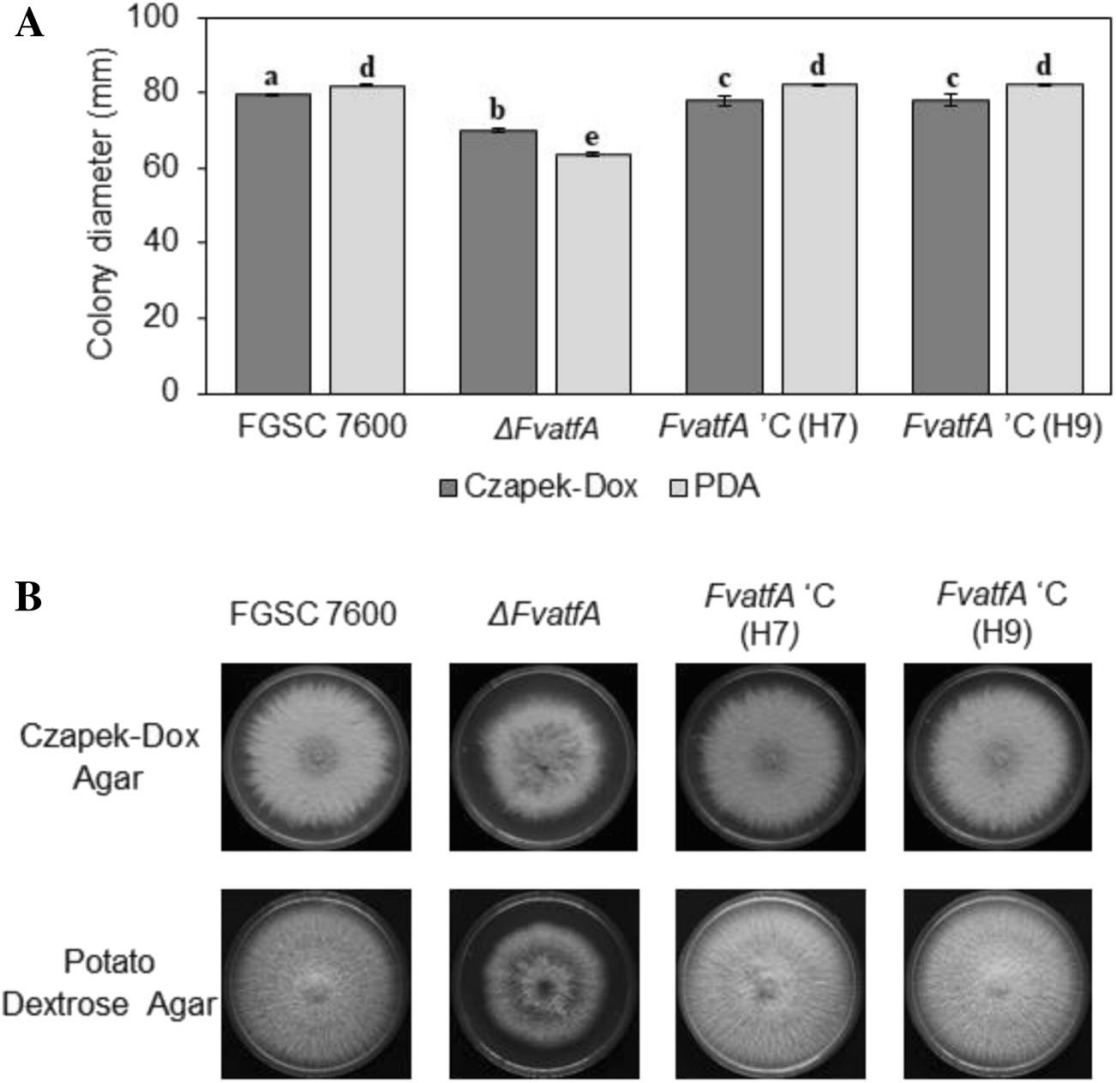
Comparison of the growth of *F. verticillioides* FGSC 7600, parental strain, its *ΔFvatfA* mutant and the *ΔFvatfA* ‘C H7- and H9-complemented strains on Czapek-Dox agar and Potato Dextrose agar. Statistical analysis of the colonial growths (part A) and one set of representative photos taken on the colonies (part B) are presented. All cultures were grown at 25 °C for 6 days. Mean colony diameter ± SD values calculated from three independent experiments are presented. Different letters indicate significant differences between strains (one-way ANOVA followed by Tukey post hoc test; adj. *p* < 0.05). For further details of the ANOVA, see Supplementary Table S5

The *ΔFvatfA* gene deletion mutant showed a decreased radial growth on both Czapek-Dox agar − 11.79%) and potato dextrose agar (− 22.16%) in comparison with the wild-type *F. verticillioides* FGSC 7600 strain, when these media were inoculated with conidia (1 × 10^5^ spores in 5 μL suspension) and the cultures were incubated at 25 °C for 6 days (Fig. 1; Supplementary Table S5).

**Fig. 2:**
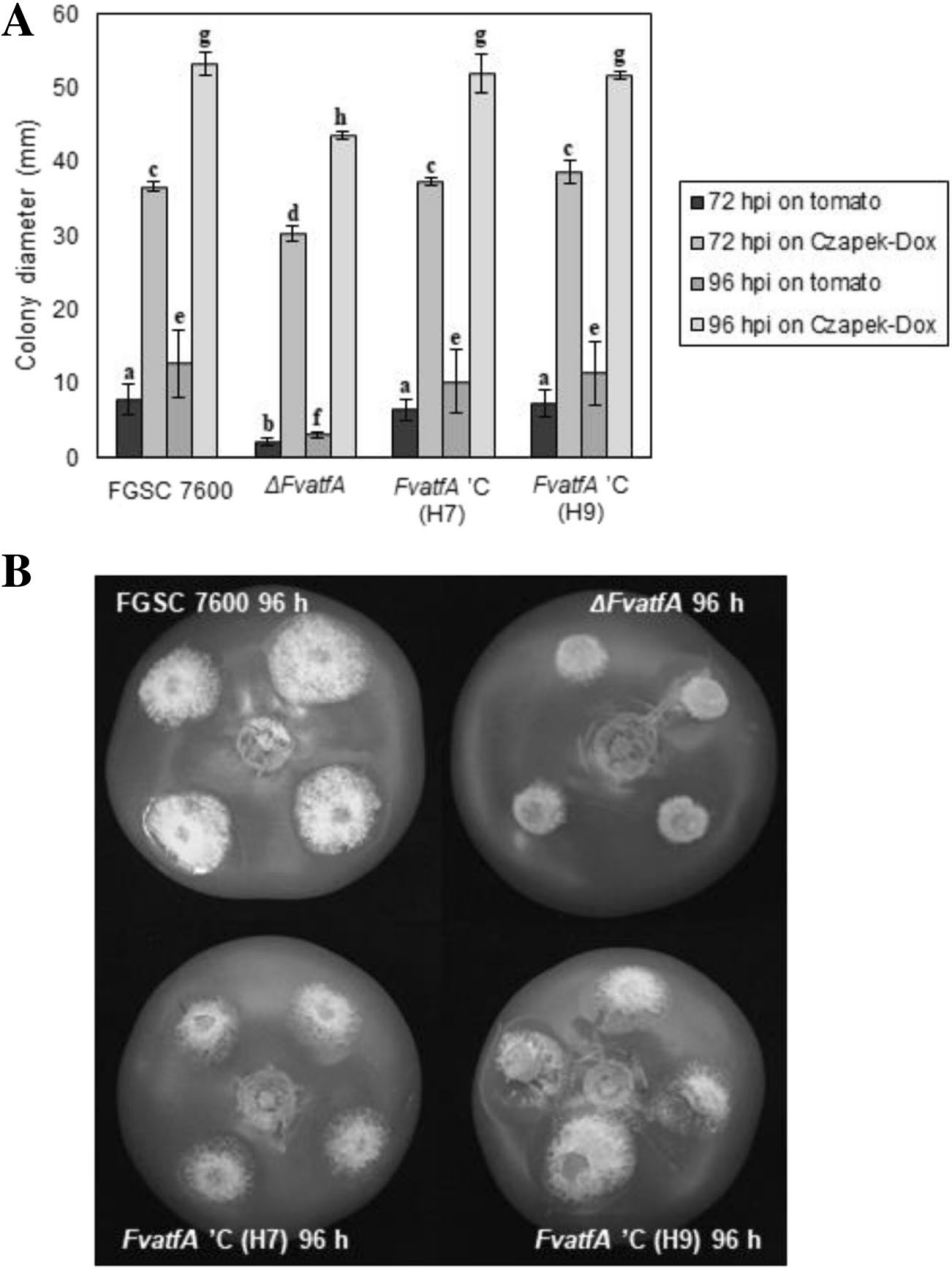
Invasive growth of fungi on tomato fruits. Plugs cut out from fungal cultures grown on Czapek-Dox agar were placed on unwounded tomato fruits and Czapek-Dox agar. Colony diameters were measured after 72 and 96 hours post-infection (hpi). Significantly reduced invasive growth was measured for the *ΔFvatfA* mutant in comparison with its wild-type parental strain, FGSC 7600 and the two *ΔFvatfA* ‘C-complemented strains, H7 and H9. Vertical bars indicate standard errors (part A). Significant differences (adj. *p* < 0.05) between strains are marked with different letters (one-way ANOVA followed by Tukey post hoc test; Supplementary Table S5). A typical set of photos taken at 96 hpi is presented on part B

Invasive growth of the wild-type, its *ΔFvatfA* mutant and the two complemented strains was compared on tomato fruits (Fig. 2; Supplementary Table S5). The average colony diameter of the wild-type strain was 7.92 and 12.75 mm, at 72 and 96 hpi, respectively, whereas the *ΔFvatfA* mutant showed significantly reduced growth (2.25 and 3.08 mm, at 72 and 96 h, respectively). The percent reduction of invasive growth of the mutant (− 71.6 and − 75.8% at 72 and 96 hpi, respectively) was much stronger than that observed on artificial media. To demonstrate this, mycelial blocks from the same colonies used for tomato infection were placed on Czapek-Dox agar and colony diameters were measured at 72 and 96 hpi. Under these conditions, the growth reduction of the *FvatfA* mutant was much less (− 17.3 and − 18.1% at 72 and 96 hpi, respectively) than that recorded on tomato fruits.

It is noteworthy that all slow-growth phenotypes observed for the *ΔFvatfA* strain were successfully complemented by re-insertion of the fully functional *FvatfA* gene into the two restored strains, H7 and H9 (Figs. 1 and 2; Supplementary Table S5).

### The *ΔFvatfA* mutant produced less and smaller spores with no influence on spore viability under heat and cold stress

**Fig. 3:**
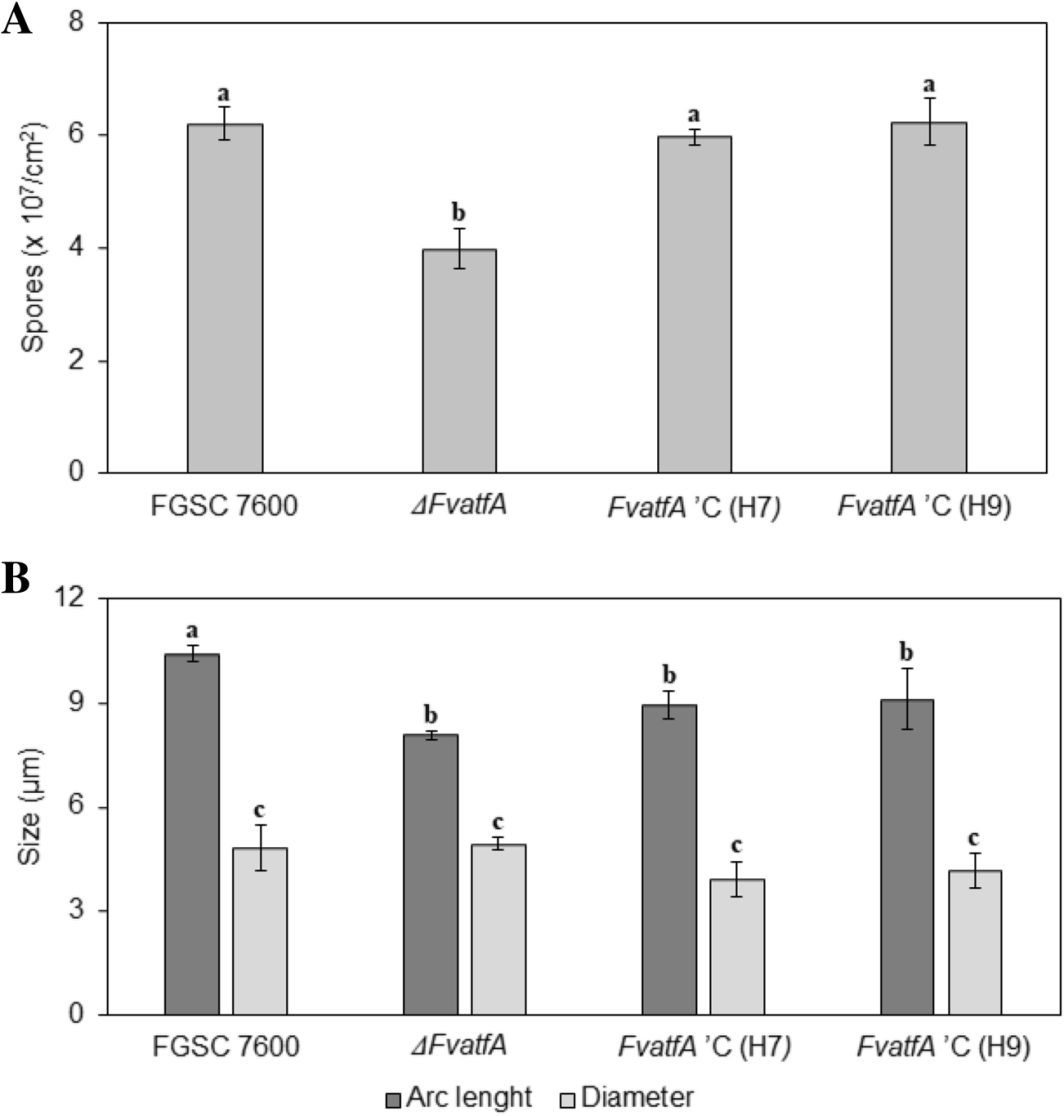
Differences in conidium production and conidium morphology among the *ΔFvatfA* mutant, its wild-type parental strain, FGSC 7600 and the two complemented strains, *ΔFvatfA* ‘C H7 and H9. In part A, spore production of the strains are presented. Agar plugs were vortexed in sterile distilled water, and spore suspensions were counted in haemocytometer. In part B, arc lengths and diameters of the spores counted with ImageJ software are presented. Significant (adj. *p* < 0.05) differences between the marked strains found in the same experiment are indicated by different letters above the columns (one-way ANOVA followed by Tukey post hoc test; Supplementary Table S5)

Conidium production of the fungi was compared on Czapek-Dox agar plates. Under these conditions, deletion of the *FvatfA* gene resulted in a weakened capability to produce microconidia. While the FGSC 7600 strain produced 6.21 ± 0.29 × 10^7^ spores cm^−2^, the *ΔFvatfA* mutant yielded 3.98 ± 0.36 × 10^7^ (i.e. 35.9% less) spores per cm^2^. Furthermore, the average arc length of the *ΔFvatfA* conidia was approximately 2 μm shorter (8.07 ± 0.13 μm) than that of the conidia produced by the wild-type strain (10.43 ± 0.23 μm), but the average width of conidia produced by the two fungi was nearly the same (4.85 ± 0.66 vs. 4.94 ± 0.18 μm for the wild-type and mutant strains, respectively) (Fig. 3; Supplementary Fig. S3; Supplementary Table S5). The arc length phenotype of the *ΔFvatfA* strain was only partially complemented in the *FvatfA* ‘C H7 and H8 strains as indicated by one-way ANOVA (Fig. 3; Supplementary Table S5).

Interestingly, deletion of *FvatfA* had no influence on heat stress (1 h exposures at 42 or 45 °C) or cold stress (7-day storage at 4 °C) tolerance of the fungus (Supplementary Fig. S3; Supplementary Table S5).

### Deletion of *FvatfA* had a negative effect on abiotic stress tolerance

**Fig. 4:**
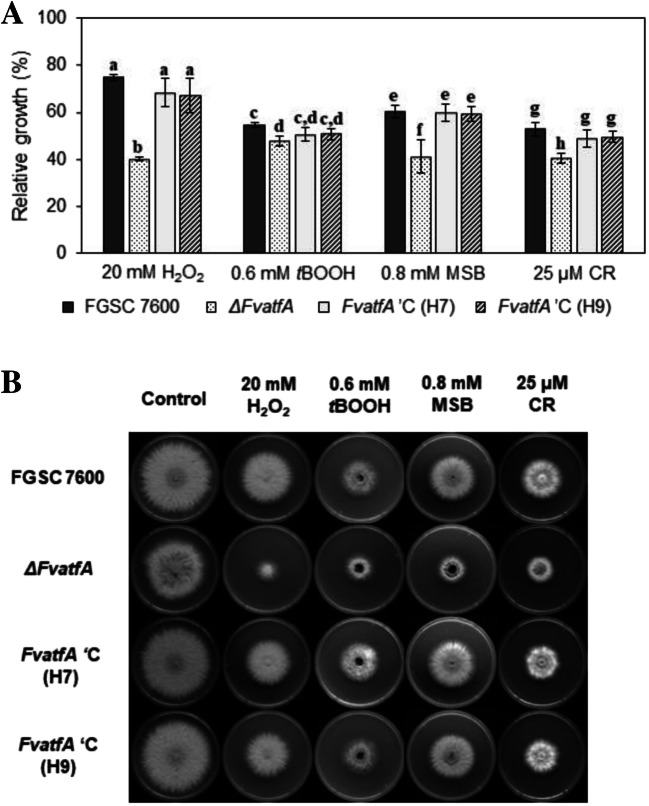
Increased oxidative (H_2_O_2_, *t*BOOH, MSB) and cell wall integrity (CR) stress sensitivity of the *ΔFvatfA* strain in comparison with its wild-type parental strain (FGSC 7600) and the two *ΔFvatfA* ‘C-complemented strains (H7, H9). Statistically increased stress sensitivities and a typical set of photos taken on stress agar plates after 6 days of incubation at 25 °C (part B) are presented. The different letters shown above the columns indicate significant (adj. *p* < 0.05) differences between the strains (one-way ANOVA followed by Tukey post hoc test; Supplementary Table S5)

The Atf1 (*S. pombe*)–AtfA (*A. nidulans*) orthologous transcription factors orchestrate the environmental stress defence in a range of fungi (Balázs et al. 2010; Hagiwara et al. 2008; Lara-Rojas et al. 2011; Nguyen et al. 2013; Qi et al. 2013; Rodrigues-Pousada et al. 2010). As expected, the *F. vertillioides ΔFvatfA* mutant possessed increased sensitivity to oxidative stress-generating agents like H_2_O_2_, *t*BOOH (initiating peroxide stresses) and MSB (causing superoxide stress) as well as to Congo Red (an elicitor of cell wall integrity stress in sensitive fungi) as calculated by one-way ANOVA using relative growth data (Fig. 4; Supplementary Table S5). The per cent decreases in stress tolerance of the *ΔFvatfA* mutant in comparison with the wild-type parental strain were as follows: 25 mM H_2_O_2_, 46.5%; 0.6 mM *t*BOOH, 12.5%; 0.8 mM MSB, 32.0%; 25 μM Congo Red, 23.6% (Fig. 4). Two-way ANOVA analysis, which was based on absolute growth values, also supported the increased sensitivity of the *ΔFvatfA* strain towards H_2_O_2_-, MSB- and Congo Red-elicited stresses (Supplementary Table S6), but the *t*BOOH-sensitive phenotype of the gene deletion mutant was not confirmed (*p* > 5%; Supplementary Table S6). Surprisingly, *ΔFvatfA* and the FGSC 7600 strain showed equal tolerance to NaCl and KCl exposures (high osmolarity stress), CdCl_2_ treatments (heavy metal stress) and diamide, responsible for glutathione/glutathione disulphide redox imbalances (Supplementary Fig. S4).

### *ΔFvatfA* is deficient in fumonisin production

**Fig. 5:**
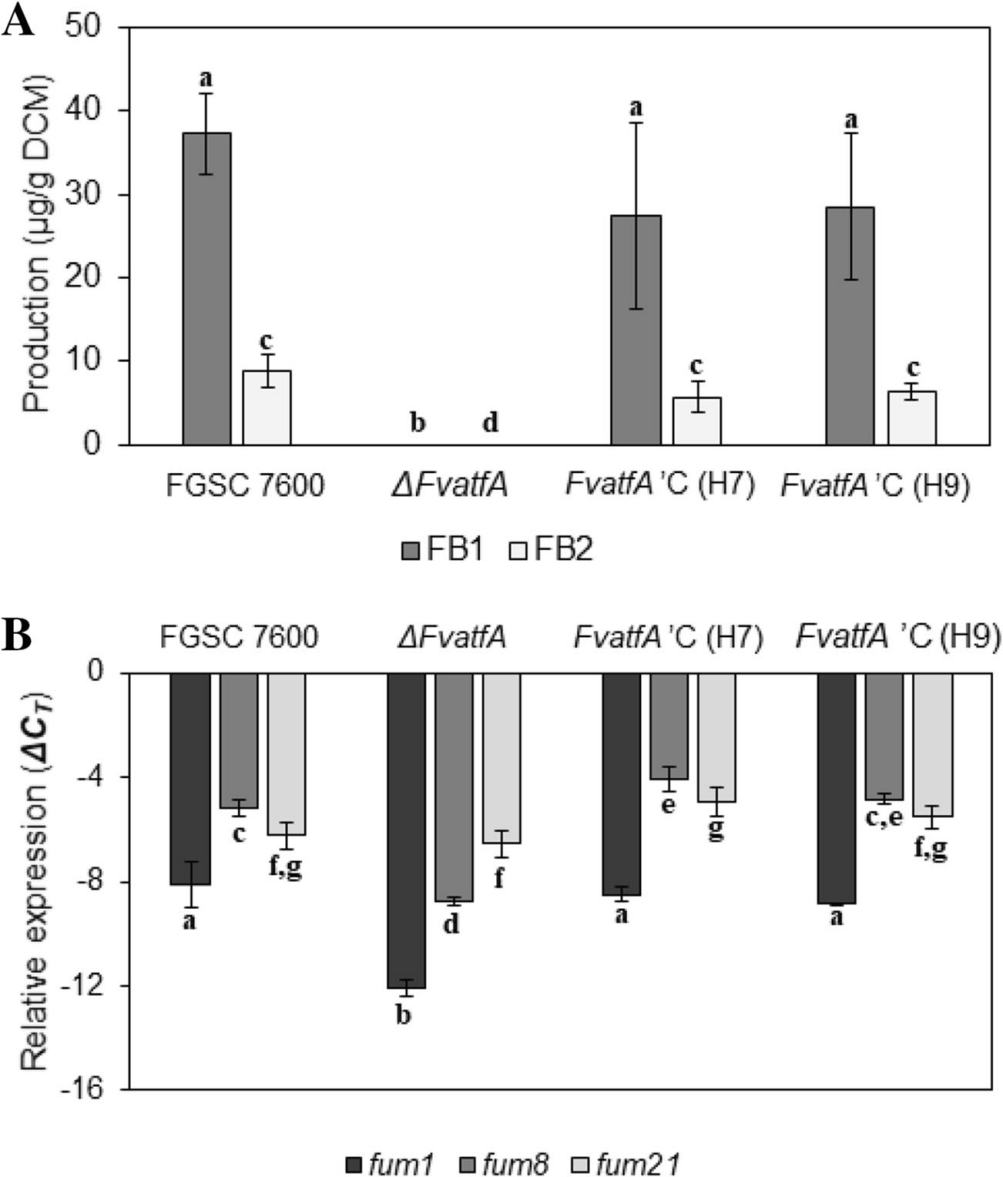
Defected fumonisin production of the *ΔFvatfA* strain. The *ΔFvatfA* gene deletion mutant secreted no fumonisin when the tested strains were cultured in Myro medium for 14 days at 25 °C. FB1 and FB2 production was measured by CE-MS in the culture fluids. In part B, expression levels of two fumonisin biosynthesis genes, *fum1* and *fum8*, as measured by qRT-PCR are shown. Significantly reduced gene expression was measured in the *ΔFvatfA* strain as compared with the wild-type parental strains and the two *ΔFvatfA* ‘C-complemented strains, H7 and H9. Expression of *fum21* (encoding a Zn(II)2Cys6-type transcriptional regulator) was not affected by the deletion of *FvatfA* (part B). Significant (adj. *p* < 0.05) differences between the marked strains in the same experiment were indicated by the same letters above the columns (one-way ANOVA, Tukey post hoc test; Supplementary Table S5)

*F. verticillioides* produces a number of mycotoxins including series B fumonisins (Blacutt et al. 2018) that can be present at biologically significant levels in maize as well as in a variety of maize-based human foodstuffs and animal feeds (Logrieco et al. 2002; Covarelli et al. 2012). In order to determine if deletion of *FvatfA* affected the production of fumonisin B1 and fumonisin B2 (FB1, FB2), the wild-type, its *ΔFvatfA* mutant and the two restored strains (H7, H9) complemented with the wild-type *FvatfA* were grown in modified Myro medium for 14 days as static cultures. In a preliminary experiment, we determined fumonisin concentrations both in the culture filtrate and the mycelial mat with CE-MS and found that 80–90% of these metabolites were present in the culture filtrate (Supplemenatry Table S7). Therefore, in subsequent experiments we measured FB1 and FB2 only in the culture filtrate. As shown in Fig. 5, deletion of *FvatfA* resulted in a drastic decrease of fumonisin production, i.e. levels of these metabolites dropped below the detection limits of CE-MS. Expression levels of two fumonisin biosynthesis genes, *fum1* and *fum8*, encoding a polyketide synthase and an *α*-oxoamine synthase, respectively, paralleled with fumonisin production: transcript levels of these two genes were significantly lower in the *ΔFvatfA* mutant, than in the wild-type and the two complemented strains. On the other hand, deletion of *FvatfA* caused no change in transcription of *fum21*, another fumonisin biosynthesis gene encoding a transcription factor with Zn(II)2Cys6 DNA-binding capability (Fig. 5; Supplementary Table S5).

### Deletion of *FvatfA* resulted in decreased carotenoid production

**Fig. 6:**
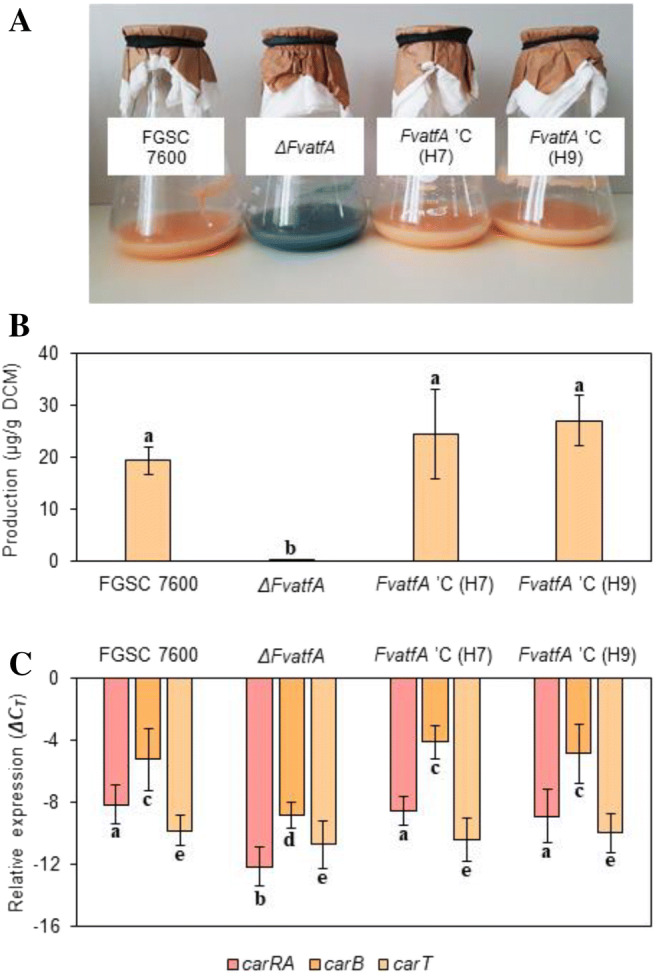
Decreased carotenoid production of the *ΔFvatfA* strain. The bluish purple colour of *ΔFvatfA* mycelia indicated an altered pigment production of the gene deletion mutant, when it was cultured in DG liquid medium for 7 days under continuous illumination (part A). In part B, the significantly decreased carotenoid content (determined spectrophotometrically) of the *ΔFvatfA* strain is presented. In part C, a significant down-regulation of *carRA* (bifunctional gene encoding phytoene synthase and carotene cyclase) and *carB* (coding for carotene desaturase) as measured by qRT-PCR is presented. Expression of *carT*, encoding the torulene cleavage enzyme, was not influenced by the deletion of *FvatfA*. The different letters above the columns indicate that the marked strains are significantly different from the others in the same experiment (adj. *p* < 0.05, one-way ANOVA followed by Tukey post hoc test). For further details, consult Supplementary Table S5

The *ΔFvatfA* mutant showed reduced yellow pigmentation in comparison with the wild-type strain when cultured on CM under continuous illumination suggesting that carotenoid biosynthesis could be influenced by deletion of the *FvatfA* gene. Fungi were incubated in liquid DG medium under continuous illumination for 7 days in shaken cultures and then carotenoids were extracted from mycelial samples and measured spectrophotometrically. As expected, only trace amounts of carotenoids were detected in cultures of the *ΔFvatfA* strain, whereas the wild type and the two restored strains produced normal amounts of these metabolites (Fig. 6; Supplementary Table S5). In *Fusarium* species, *carRA* (encoding a bifunctional enzyme with phytoene synthase and carotene cyclase activities), *carB* (coding for carotene desaturase) and *carT* (encoding carotene-cleaving oxygenase) play an important role in carotenoid biosynthesis (Ádám et al. 2011). We compared, therefore expression levels of the *carRA*, *carB* and *carT* genes in the wild-type, the *ΔFvatfA* mutant and the two complemented strains using qRT-PCR. RNA was isolated from mycelial samples of cultures grown for 4 days in the dark and then illuminated for 2 h. The relative expression of *carRA* and *carB* was significantly lower in the *ΔFvatfA* mutant than in the wild-type parental strain and the two restored strains (H7, H9) indicating that down-regulation of these two genes in the *ΔFvatfA* mutant is the cause of decreased carotenoid production. On the contrary, deletion of *FvatfA* had no effect on *carT* expression (Fig. 6; Supplementary Table S5).

### Bikaverin biosynthesis increased in the *ΔFvatfA* mutant

**Fig. 7:**
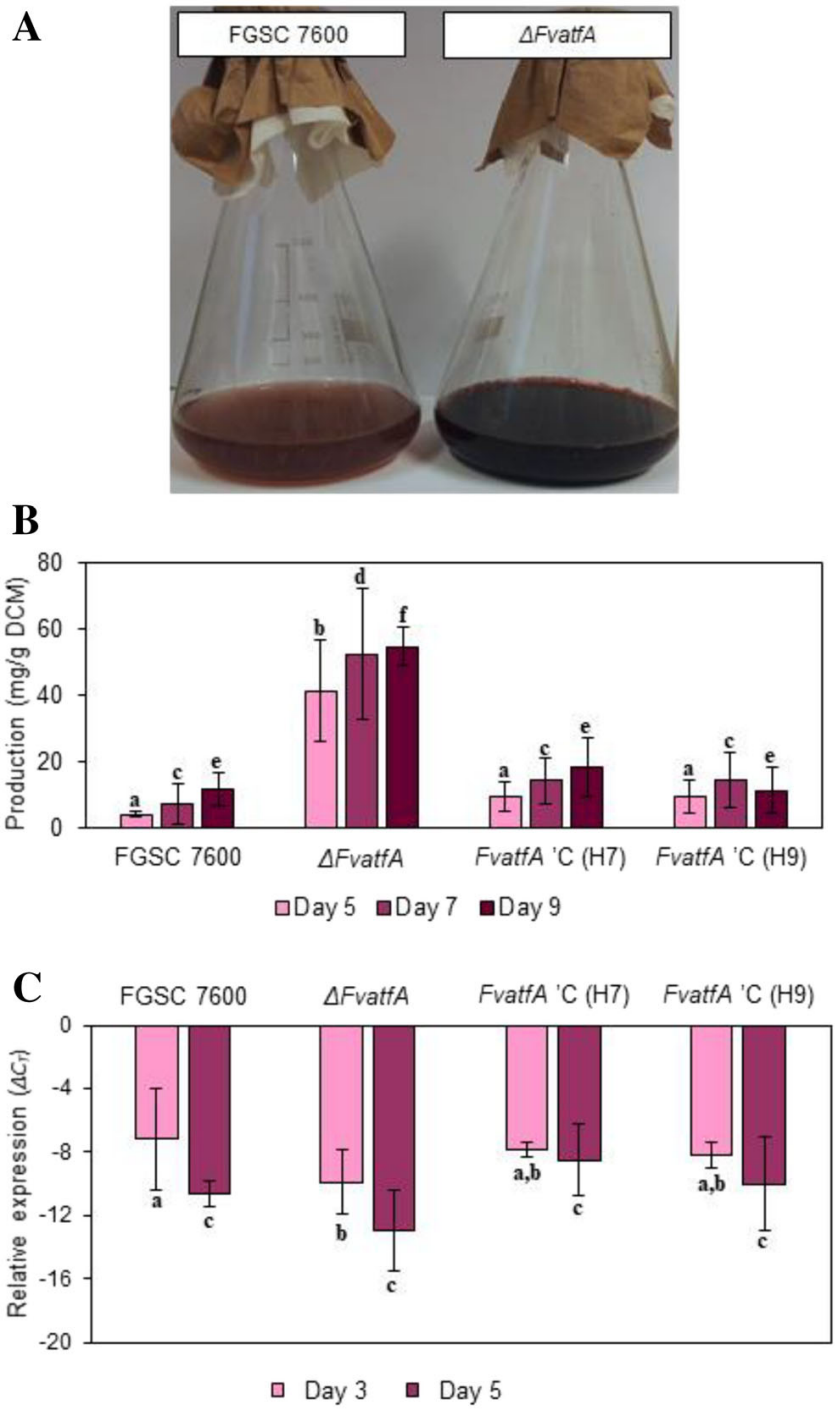
Increased bikaverin production by the *ΔFvatfA* mutant in shaken liquid cultures. Visual comparison of the cultures (part A, after 9 days of incubation) indicated a massive overproduction of this polyketide pigment by the gene-deletion mutant. This was confirmed by spectrophotometric measurement of bikaverin production. Deletion of *FvatfA* had no effect on the expression of *bik1*, encoding the bikaverin polyketide synthase as measured by qRT-PCR (part C). The different letters in the same experiment above the columns mean that the marked strains differed significantly (adj. *p* < 0.05) from each other in pairwise comparisons (one-way ANOVA followed by Tukey post hoc test). For further details, see Table S5

The *ΔFvatfA* mutant showed an intense reddish-purple pigmentation on Czapek-Dox agar plates after 7 days of incubation, a phenotype, not observed in the wild-type strain grown under similar conditions. As bikaverin is the major red pigment produced by *Fusarium* species in culture (Linnemannstöns et al. 2002a), we presumed that this metabolite is responsible for the deep red colour of the *ΔFvatfA* mutant and, therefore measured bikaverin production of the fungi involved in this study according to the protocol of Bell et al. (2003). It is noteworthy that the *ΔFvatfA* mutant produced approximately 10 times more bikaverin than the wild-type and the restored strains, H7 and H9 after 5 days of incubation, but incubation, extended for 7–9 days resulted in no further increase in bikaverin yield (Fig. 7; Supplementary Table S6). Expression of *bik1* (formerly *pks4*), a key polyketide synthase gene, responsible for bikaverin synthesis in *F. verticillioides* (Linnemannstöns et al. 2002a) showed, however, no increase (or even a slight down-regulation at 3 days of incubation) in the *ΔFvatfA* mutant in comparison with the FGSC 7600 control strain, indicating that the cause(s) of overproduction of this metabolite are others than overexpression of *bik1*.

### In silico promoter analysis

The ATF/CREB bZIP transcription factors are known to bind to the consensus sequence, TGACGTCA (Loeken 1993; Kvietikova et al. 1995; Sakamoto et al. 2008; Hong et al. 2013) and, therefore, we performed an in silico promoter analysis to trace the presence of this motif on sequences of the secondary metabolite genes subjected to gene expression measurements in this study. As summarized in Supplementary Table S4, all genes we found to be down-regulated in the *ΔFvatfA* mutant, including *fum1, fum8, carRA*, *carB* and *bik1* (Figs. 5, 6 and 7), contained at least one putative ATF/CREB binding site in their promoters between the 5’-end of the intergenic region and + 50 bp down-stream of TSS (all predicted intergenic regions for the tested genes were < 1000 bp; Wolf et al. 2016), whereas the two other genes (*fum21*, *carT*), whose expression was not affected in the *ΔFvatfA* mutant (Figs. 5 and 6), either lacked such sequence (*fum21*) or possessed two (*carT*) on its putative promoter region (Supplementary Fig. S6; Supplementary Table S4). Importantly, further putative ATF/CREB binding motifs were identified in the 5’-untranslated regions of all tested genes (Supplementary Fig. S6; Supplementary Table S4).

### Overall characterization of the strains

PCA clearly separated the *ΔFvatfA* mutant from both the wild-type parental strain, FGSC 7600 and the two genetically complemented strains (*FvatfA* ‘C H7, H9; Supplementary Fig. S5; Supplementary Table S8). This spectacular separation of the *ΔFvatfA* mutant from the other three strains resulted from differences in a number of phenotypic features including vegetative growth, invasive growth, spore production, abiotic stress tolerance and secondary metabolite production (Supplementary Table S8). These findings underline the pleiotropic effects of deletion of *FvatfA.* Interestingly, some levels of separation also appeared between the wild-type parental strain, FGSC 7600 and the two complemented strains, *FvatfA* ‘C H7 and H9 due to the minor differences found in their spore morphology and viability (Supplementary Fig. S5; Supplementary Table S8).

## Discussion

The *ΔFvatfA* gene deletion mutant constructed in this study greatly differed in its phenotype from the wild type parental strain shedding light on the versatile physiological functions played by this bZIP transcription factor in the orchestration of vegetative and invasive growths, asexual development, environmental stress tolerance and secondary metabolite production in *F. verticillioides* (Supplementary Table S5). PC analysis of the phenotypes also supported the pleiotropic nature of *FvatfA* (Supplementary Fig. S5; Supplementary Table S8) similarly to other *FvatfA* orthologs functionally characterized thus far (Takeda et al. 1995; Hagiwara et al. 2009, 2016; Balázs et al. 2010; Guo et al. 2010; Sansó et al. 2011; Lara-Rojas et al. 2011; Roze et al. 2011; Temme et al. 2012; Hong et al. 2013; Qi et al. 2013; Nguyen et al. 2013; Jiang et al. 2015; Wee et al. 2017). Important to note that complementation of the *ΔFvatfA* strain with a fully functional *FvatfA* gene successfully restored the wild-type phenotypes in strains *FvatfA* ‘C H7 and H9 (Supplementary Table S5); PC analysis placed these strains close to the wild-type parental strain FGSC 7600 confirming this statement (Supplementary Fig. S5; Supplementary Table S8). The minor phenotypic differences observed between the wild type parental strain and the *FvatfA* ‘C H7- and H9-complemented strains, (Supplementary Fig. S5; Supplementary Table S8) are normal and reflect (i) either variations in the environment of the *FvatfA* gene after a random insertion of the *FvatfA* complementation cassette into the genome of the gene deletion strain or (ii) some kinds of microevolutionary processes working in filamentous fungi (Ballard et al. 2018, 2019) especially under the selection pressure exerted by protoplasting and transformation.

In details, the *ΔFvatfA* strain showed slightly reduced growth on both Czapek-Dox (− 11.79%) and potato dextrose (− 22.16%) agars (Fig. 1; Supplementary Table S5) in harmony with previous observations on the *ΔMoatf1* mutant of *M. oryzae* (Guo et al. 2010) and the *ΔFgatf1* strain of *F. graminearum* (Nguyen et al. 2013; Jiang et al. 2015). As the regulatory effects of AtfA orthologs on cell wall biogenesis and primary metabolism have previously been demonstrated in *B. cinerea* (Temme et al. 2012) and *A. nidulans* (Emri et al. 2015; Orosz et al. 2017) we can assume that deletion of *FvatfA* also led to disturbances in basic metabolic and physiological processes, and this was the cause of the observed growth reduction in *F. verticillioides.*

The *ΔFvatfA* mutant showed greatly reduced colonizing capability on unwounded tomato fruits in comparison with the wild-type and the *ΔFvatfA* ‘C-complemented strains (− 71.6 and − 75.8%, at 72 and 96 hpi, respectively; Fig. 2; Supplementary Table S5). This debilitation in invasive growth suggests that FvAtfA contributes to virulence in *F. verticillioides* similarly to previous findings in experiments with other plant pathogenic fungi (*C. purpurea* on rye (Nathues et al. 2004), *M. oryzae* on rice (Guo et al. 2010), *F. oxysporum* on Cavendish banana (Qi et al. 2013), *F. graminearum* on wheat, maize *Brachypodium distachyon* (Nguyen et al. 2013; Jiang et al. 2015)}.

The elimination of Atf1/AtfA ortholog transcription factors also disturbed both asexual and sexual reproduction in many plant pathogenic fungi. The *Δbcatf1* mutant of *B. cinerea* was impaired in conidiation and sclerotium production (Temme et al. 2012) and the *ΔFgatf1* strains of *F. graminearum* were also weakened in conidium yields and their sexual reproduction was delayed, too (Nguyen et al. 2013; Jiang et al. 2015). Similarly to the *ΔFgatf1* strains of *F. graminearum*, the *ΔFvatfA* mutant produced 35.9% less conidia than the wild-type parental strain and conidia of the mutant had significantly shorter arc lengths (Fig. 3; Supplementary Fig. S3). Reduction in the arc length of conidia has been reported previously for the *ΔFoatf1* mutant of *F. oxysporum* (Qi et al. 2013).

Since the Atf1/AtfA ortholog transcription factors are typically centerpieces of the environmental stress defence systems of fungi furnished with this type of bZIP domain proteins (Gasch 2007; Chen et al. 2003; Sansó et al. 2008, 2011; Hagiwara et al. 2008, 2009; Balázs et al. 2010; Lara-Rojas et al. 2011), the enhanced sensitivity of the *ΔFvatfA* mutant to oxidative (H_2_O_2_, MSB) and cell wall integrity (Congo Red) stress (Fig. 4; Supplementary Table S5) was foreseeable. It is noteworthy that meanwhile one-way ANOVA analysis of relative growth data also indicated a *t*BOOH-sensitive phenotype of the gene deletion strain two-way ANOVA analysis of colony diameters of *t*BOOH-exposed cultures did not confirm this phenotype (Fig. 4; Supplementary Table S6). Furthermore, the tolerance of the mutant to high-osmolarity (NaCl, KCl, sorbitol), heavy metal (CdCl_2_) and glutathione redox imbalance (diamide) stress was similar to that of the wild-type and the complemented strains (Supplementary Fig. S4).

Previous studies with other plant pathogenic fungi revealed diverse stress sensitivity phenotypes for other Atf1/AtfA ortholog deficient strains. Increased oxidative stress sensitivity was observed in *atf1*/*atfA* orthologous gene disruption mutants of *M. oryzae* (H_2_O_2_; Guo et al. 2010) and *F. graminearum* (H_2_O_2_; Jiang et al. 2015), enhanced cell wall integrity stress sensitivity was demonstrated in similar mutants of *B. cinerea* (Congo Red, calcofluor white; Temme et al. 2012) and *F. graminearum* (Congo Red; Jiang et al. 2015) and decreased high-osmolarity stress tolerance was found in *FvatfA* mutants of *F. graminearum* (NaCl; Nguyen et al. 2013; Jiang et al. 2015). The orchestration of environmental stress defence by Atf1/AtfA orthologs seems to be especially useful when fungal pathogens try to attenuate the response (e.g. oxidative burst) of their host plants (Nathues et al. 2004; Guo et al. 2010; Qi et al. 2013; Nguyen et al. 2013; Jiang et al. 2015).

The elevated oxidative stress (H_2_O_2_, *t*BOOH, MSB, diamide) sensitivity was not accompanied by high-osmolarity stress (NaCl) sensitivity in the *ΔatfA* mutant of *A. nidulans* either (Balázs et al. 2010; Emri et al. 2015), although deletion of *atfA* elicited global transcriptional changes under NaCl exposure (Emri et al. 2015). The unpredictable occurrence of some unusual stress sensitivity phenotypes in Atf1/AtfA ortholog deficient fungal strains can be explained by the complexity, flexibility and robustness of the stress response systems operating in filamentous fungi (Miskei et al. 2009; Emri et al. 2018). The abundance of some important stress-activated and mitogen-activated protein kinases, response regulators, transcription factors in the stress response regulatory networks foreshadows the likely existence of efficient compensatory mechanisms for the loss of any of these stress response elements (Miskei et al. 2009). The high-complexity stress responses orchestrated by Atf1/AtfA orthologs can also be associated with (i) the impact of these transcription factors on the expression of other important elements of the stress signaling and stress response regulatory network, (ii) direct, physical interactions of the Atf1/AtfA orthologs with elements of this network (e.g. with other bZIP-type transcription factors) as well as (iii) the interplays and cross-talks between AtfA-dependent stress responses (Orosz et al. 2017).

While AtfA orthologous transcription factors play a crucial role in the stress defence of both the developing and the dormant conidia of aspergilli (Hagiwara et al. 2008, 2009, 2014, 2016; Sakamoto et al. 2009; Balázs et al. 2010; Lara-Rojas et al. 2011), deletion of *FvatfA* had no influence on heat and cold stress tolerance of the asexual spores in *F. verticillioides* (Supplementary Fig. S3; Supplementary Table S5). This finding indicates that the primary stress response-related function of FvAtfA is the orchestration of stress defence in the mycelial tissue of this fungus without safeguarding the survival of asexual spores.

One of the most important observations in this study was the inability of the *ΔFvatfA* mutant to produce fumonisins in Myro medium (Fig. 5). The abolishment of mycotoxin production was coupled to and explained with a significant down-regulation of two fumonisin biosynthesis genes, *fum1* (encoding the fumonisin polyketide synthase) and *fum8* (coding for α-oxoaminesynthase) (Fig. 5). Interestingly, no down-regulation of *fum21* was observed in the *ΔFvatfA* mutant. This gene codes for a Zn(II)2Cys6 binuclear cluster DNA-binding domain containing transcription factor and is considered a local positive regulator within the fumonisin biosynthetic gene cluster (Brown et al. 2007). The results of promoter analysis of the tested genes were in line with changes in the gene expression levels. The promoters of *fum1* and *fum8* contained a putative CRE sequence meanwhile no such sequence was identified in the promoter region of *fum21* (between the 5’-end of the intergenic region and position + 50 bp down-stream of TSS; Supplementary Fig. S6; Supplementary Table S4). Further research should aim at demonstrating the hypothesized physical interactions between FvAtfA and these putative elements using either gel electrophoresis mobility shift assay (EMSA) or chromatin immunoprecipitation sequencing (ChIP-seq) (Roze et al. 2011; Hong et al. 2013; Wee et al. 2017). In *Aspergillus parasiticus*, seven genes in the aflatoxin biosynthetic gene cluster with CRE in their promoters were under direct AtfB regulation meanwhile other promoters lacking CRE were not recognised by this bZIP transcription factor (Roze et al. 2011). It is worth noting that although the position preferences of bZIP transcription factors are from − 100 to − 50 bp with respect to TSS (*Arabidopsis thaliana*; Yu et al. 2016), the possible recognition of other down-stream ATF/CREB transcription factor binding sites by FvAtfA should also be considered in future studies (Supplementary Fig. S6; Supplementary Table S4; Roze et al. 2011; Hong et al. 2013).

Although SakA/HogA MAPK physically interacts with AtfA in *A. nidulans* (Lara-Rojas et al. 2011) future research is needed on MAPK(s) functioning up-stream of the Atf1/AtfA homologs. Interestingly, Kohut et al. (2009) found temporary up-regulation of *fum1* and *fum8* followed by a rapid onset of fumonisin production in a *Fusarium proliferatum ΔFphog1* gene deletion mutant, under nitrogen starvation. They explained the early up-regulation of fumonisin biosynthesis genes in the mutant by the increased sensitivity of the *ΔFphog1* strain to N-starvation stress in the absence of functional Hog-1 MAPK pathway. On the other hand, deletion of *Fvmk1* encoding for Fus3/Kss1 MAPK in *F. verticillioides* resulted in decreased expression of *fum1* and *fum8* and reduced production of fumonisin (Zhang et al. 2011), similarly to the *ΔFvatfA* mutant as demonstrated in this study (Fig. 5). It is important to note that both positive and negative cross-talks exist between Fmk1, Mpk1 and Hog1 MAPKs during stress adaptation as described in *F. oxysporum* (Segorbe et al. 2017). This means that stress response regulatory transcription factors like FvAtfA may receive signals from various signal transduction routes depending on species and environmental conditions.

Deletion of *F. graminearum Fgatf1*, a closely related ortholog of *F. verticillioides FvatfA* (Supplementary Table S1; Supplementary Fig. S1), also affected environmental stress defence and secondary metabolite (deoxynivalenol, zearalenone) production (Nguyen et al. 2013; Jiang et al. 2015). All these observations are indicative of the inherently coupled co-regulation of stress defence and secondary metabolite production in fusaria.

In *A. parasiticus*, an aflatoxin producing fungus a complex regulatory network of various transcription factors (AtfB, SrrA, AP-1 and MsnA, where AtfB and AP-1 are bZIP-type transcription factors) is likely to coordinate both oxidative stress response and secondary metabolism (Roze et al. 2011; Hong et al. 2013). This regulatory network seems to receive and integrate signals elicited by the cAMP-protein kinase A (down-regulated by oxidative stress) and stress-activated protein kinase pathways (Hong et al. 2013). Similar regulatory network may operate in *Fusarium* spp. as well.

*F. verticillioides ΔFvatfA,* debilitated in fumonisin production is a disarmed strain and, therefore it may open the way to the development of new-type biology-based mycotoxin control strategies including RNAi-mediated gene silencing technologies (McDonald et al. 2005; Majumdar et al. 2017; Pareek and Rajam 2017; Johnson et al. 2018) targeting *FvatfA* and/or other regulators of mycotoxin biosynthesis.

*Fusarium* spp. can also produce a plethora of valuable carotenoids including β-carotene, lycopene and neurosporaxantin (Prado-Cabrero et al. 2007, 2009; Avalos et al. 2012; Gmoser et al. 2017). As shown in Fig. 6, deletion of *FvatfA* decreased the carotenoid production of *F. verticillioides* considerably, paralleled with the down-regulation of two carotenoid biosynthesis genes, *carRA* and *carB* (Linnemannstöns et al. 2002b; Prado-Cabrero et al. 2007; Avalos et al. 2017), which also contained putative ATF/CREB binding sites at their promoters (Supplementary Fig. S6; Supplementary Table S4). Similar down-regulation of carotenoid biosynthesis was observed by Ádám et al. (2011) in *ΔFvMAT1-2-1* knockout mutants of *F. verticillioides*, indicating a possible interplay between FvAtfA and mating type genes in the regulation of carotenogenesis. Future studies in this field are foreseen to focus on the overproduction of AtfA-type transcription factors in *Fusarium* species to see whether this kind of genetic manipulation could result in carotenoid overproduction strains. The light-induced appearance of an unusual bluish purple color as detected in *ΔFvatfA* cultures (Fig. 6) may lead to the discovery of an until unknown *Fusarium* pigment. Further studies should also aim at shedding light on the insensitivity of the torulene cleavage enzyme encoding *carT* gene to *FvatfA* deletion despite of the putative CRE sequences present on its promoter (Supplementary Fig. S6; Supplementary Table S4).

These novel pieces of information on the regulation of pigment production in *F. verticillioides* might also be utilized in *Fusarium*-based mycoprotein production as the antioxidant and antihyperlipidemic properties of the fungal biomass certainly improve the biological value of such products (Thomas et al. 2017). It is noteworthy that beneficial health, e.g. antiatherosclerotic effects of fungal carotenoids have been reported in animal models (Setnikar et al. 2005; Kumar et al. 2011).

*F. verticillioides* also produces a red coloured secondary metabolite called bikaverin with potent antibacterial (Deshmukh et al. 2014; Sondergaard et al. 2016; Lebeau et al. 2019), antiprotozoan (Balan et al. 1970), antioomycete (Son et al. 2008) and antitumor (Fuska et al. 1975; Zhan et al. 2007; Limón et al. 2010) activities. The nearly tenfold overproduction of bikaverin by the *ΔFvatfA* strain in a submerged liquid culture (Fig. 7) may also attract the attention of industrial experts. In previous studies (Deshmukh and Purohit 2014), siRNA mediated silencing of *hmgR*, encoding hydroxymethyl glutaryl coenzyme A reductase in the carotenoid and gibberellin biosynthesis pathways in *Fusarium* sp. HKF15, resulted in a 41% increase of the bikaverin yield paralleled with a significant up-regulation of *bik1*, the bikaverin polyketide synthase gene (Wiemann et al. 2009). The *bik1* gene also possesses putative ATF/CREB binding sites on its promoter (Supplementary Fig. S6; Supplementary Table S4) and, not surprisingly, the deletion of *FvatfA* decreased the expression of *bik1* at least at 3 days (but not at 5 days) incubation (Fig. 7). The obvious contradiction between the tremendous increase in bikaverin production measured in the gene disruption mutant and the down-regulation (or the absence of up-regulation) of *bik1* can only be explained with the drastic down-regulation of both the fumonisin and the carotenoid pathways observed in the absence of FvatfA. Due to disturbances in fumonisin and carotenoid biosynthesis, the building blocks needed for these metabolites became redundant and were probably channelled towards the synthesis of other metabolites including bikaverin (Fig. 7) and a still unidentified bluish purple pigment that appeared in illuminated cultures (Fig. 6). Furthermore, expression levels of *bik1* were basically high in the wild type, as the medium we used for testing bikaverin production was a bikaverin inducing one (Bell et al. 2003) allowing not much further up-regulation of this gene, if any in the *ΔFvatfA* mutant. Deletion of *atf1/atfA* orthologs in *B. cinerea* (Temme et al. 2012) and *A. nidulans* (Emri et al. 2015; Orosz et al. 2017) also had a definite impact on primary and secondary metabolism, confirming the findings of the present research.

Further studies are needed to elucidate how the Atf1/AtfA orthologous transcription factors fit into the complex regulatory network coordinating the production of bikaverin (and also other pigments like aurofusarin) in fusaria (Wiemann et al. 2009; Studt et al. 2013; Nguyen et al. 2013; Niehaus et al. 2017). Interplays with elements of carbon (Choi and Xu 2010; Kohut et al. 2010; García-Martínez et al. 2012; Studt et al. 2013), nitrogen (Teichert et al. 2006; Wagner et al. 2010; Pfannmüller et al. 2017), pH (Wiemann et al. 2009) and light- responsive (Castrillo et al. 2013)signalling and regulatory pathways, with constituents of velvet-like (Wiemann et al. 2010) and SAGA (Rösler et al. 2016) complexes as well as with other developmental regulators (Niehaus et al. 2017) and gene products (Choi et al. 2008) are foreseeable since elimination of these elements have also resulted in bikaverin overproduction in various *Fusarium* spp.

## Electronic supplementary material

ESM 1 (PDF 1.33 mb)

## References

[CR1] Ádám AL, García-Martínez J, Szűcs EP, Avalos J, Hornok L (2011). The *MAT1-2-1* mating-type gene upregulates photo-inducible carotenoid biosynthesis in *Fusarium verticillioides*. FEMS Microbiol Lett.

[CR2] Alberts JF, van Zyl WH, Gelderblom WC (2016). Biologically based methods for control of fumonisin-producing *Fusarium* species and reduction of the fumonisins. Front Microbiol.

[CR3] Antal K, Gila CB, Pócsi I, Emri T (2019) General stress response or adaptation to rapid growth in *Aspergillus nidulans*? Fungal Biol (in press). 10.1016/j.funbio.2019.10.009 10.1016/j.funbio.2019.10.009 32389300

[CR4] Avalos J, Prado-Cabrero A, Estrada AF (2012). Neurosporaxanthin production by *Neurospora* and *Fusarium*. Methods Mol Biol.

[CR5] Avalos J, Pardo-Medina J, Parra-Rivero O, Ruger-Herreros M, Rodríguez-Ortiz R, Hornero-Méndez D, Limón MC (2017). Carotenoid biosynthesis in *Fusarium*. J Fungi.

[CR6] Balan J, Fuska J, Kuhr I, Kuhrová V (1970). Bikaverin, an antibiotic from *Gibberella fujikuroi*, effective against *Leishmania brasiliensis*. Folia Microbiol.

[CR7] Balázs A, Pócsi I, Hamari Z, Leiter É, Emri T, Miskei M, Oláh J, Tóth V, Hegedűs N, Prade RA, Molnár M, Pócsi I (2010). AtfA bZIP-type transcription factor regulates oxidative and osmotic stress responses in *Aspergillus nidulans*. Mol Genet Genomics.

[CR8] Ballard E, Melchers WJG, Zoll J, Brown AJP, Verweij PE, Warris A (2018). In-host microevolution of *Aspergillus fumigatus*: a phenotypic and genotypic analysis. Fungal Genet Biol.

[CR9] Ballard E, Zoll J, Melchers WJG, Brown AJP, Warris A, Verweij PE (2019). Raw genome sequence data for 13 isogenic *Aspergillus fumigatus* strains isolated over a 2 year period from a patient with chronic granulomatous disease. Data Brief.

[CR10] Barnett LMA, Cummings BS (2018). Nephrotoxicity and renal pathophysiology: a contemporary perspective. Toxicol Sci.

[CR11] Bell AA, Wheeler MH, Liu J, Stipanovic RD, Puckhaber LS, Orta H (2003). United States Department of Agriculture — Agricultural Research Service studies on polyketide toxins of *Fusarium oxysporum* f sp *vasinfectum*: potential targets for disease control. Pest Manag Sci.

[CR12] Blacutt AA, Gold SE, Voss KA, Gao M, Glenn AE (2018). *Fusarium verticillioides*: advancements in understanding the toxicity, virulence, and niche adaptations of a model mycotoxigenic pathogen of maize. Phytopathology.

[CR13] Brown DW, Butchko RA, Busman M, Proctor RH (2007). The *Fusarium verticillioides FUM* gene cluster encodes a Zn(II)2Cys6 protein that affects *FUM* gene expression and fumonisin production. Eukaryot Cell.

[CR14] Butchko RA, Brown DW, Busman M, Tudzynski B, Wiemann P (2012). *Lae1* regulates expression of multiple secondary metabolite gene clusters in *Fusarium verticillioides*. Fungal Genet Biol.

[CR15] Castrillo M, García-Martínez J, Avalos J (2013). Light-dependent functions of the *Fusarium fujikuroi* CryD DASH cryptochrome in development and secondary metabolism. Appl Environ Microbiol.

[CR16] Chelkowski J, Zajkowski P, Visconti A (1992). Bikaverin production by *Fusarium* species. Mycotoxin Res.

[CR17] Chen D, Toone WM, Mata J, Lyne R, Burns G, Kivinen K, Brazma A, Jones N, Bähler J (2003). Global transcriptional responses of fission yeast to environmental stress. Mol Biol Cell..

[CR18] Choi YE, Xu JR (2010). The cAMP signaling pathway in *Fusarium verticillioides* is important for conidiation, plant infection, and stress responses but not fumonisin production. MPMI.

[CR19] Choi YE, Brown JA, Williams CB, Canales LL, Shim WB (2008). *GAC1*, a gene encoding a putative GTPase-activating protein, regulates bikaverin biosynthesis in *Fusarium verticillioides*. Mycologia.

[CR20] Chomczynski P (1993). A reagent for the single-step simultaneous isolation of RNA, DNA and proteins from cell and tissue samples. Biotechniques.

[CR21] Covarelli L, Stifano S, Beccari G, Raggi L, Lattanzio VMT, Albertini E (2012). Characterization of *Fusarium verticillioides* strains isolated from maize in Italy: fumonisin production, pathogenicity and genetic variability. Food Microbiol.

[CR22] Deshmukh R, Purohit HJ (2014). siRNA mediated gene silencing in *Fusarium* sp. HKF15 for overproduction of bikaverin. Bioresour Technol.

[CR23] Deshmukh R, Mathew A, Purohit HJ (2014). Characterization of antibacterial activity of bikaverin from *Fusarium* sp. HKF15. J Biosci Bioeng.

[CR24] Di Pietro A, García-Maceira I, Méglecz E, Roncero MIG (2001). A MAP kinase of the vascular wilt fungus *Fusarium oxysporum* is essential for root penetration and pathogenesis. Molecular Microbiology.

[CR25] Emri T, Szarvas V, Orosz E, Antal K, Park H, Han KH, Yu JH, Pócsi I (2015). Core oxidative stress response in *Aspergillus nidulans*. BMC Genomics.

[CR26] Emri T, Antal K, Riley R, Karányi Z, Miskei M, Orosz E, Baker SE, Wiebenga A, de Vries RP, Pócsi I (2018). Duplications and losses of genes encoding known elements of the stress defence system of the Aspergilli contribute to the evolution of these filamentous fungi but do not directly influence their environmental stress tolerance. Stud Mycol.

[CR27] Farré D, Roset R, Huerta M, Adsuara JE, Roselló L, Albà MM, Messeguer X (2003). Identification of patterns in biological sequences at the ALGGEN server: PROMO and MALGEN. Nucleic Acids Res.

[CR28] Fuska J, Proksa B, Fusková A (1975). New potential cytotoxic and antitumor substances I. In vitro effect of bikaverin and its derivatives on cells of certain tumors. Neoplasma.

[CR29] García-Martínez J, Ádám AL, Avalos J (2012). Adenylyl cyclase plays a regulatory role in development, stress resistance and secondary metabolism in *Fusarium fujikuroi*. PLoS One.

[CR30] Gasch AP (2007). Comparative genomics of the environmental stress response in ascomycete fungi. Yeast.

[CR31] Gil-Serna J, Vázquez C, Patiño B (2019) Genetic regulation of aflatoxin, ochratoxin A, trichothecene, and fumonisin biosynthesis: a review. Int Microbiol doi. 10.1007/s10123-019-00084-2 10.1007/s10123-019-00084-2 31144067

[CR32] Gmoser R, Ferreira JA, Lennartsson PR, Taherzadeh MJ (2017). Filamentous ascomycetes fungi as a source of natural pigments. Fungal Biol Biotechnol.

[CR33] Gu Q, Wang Z, Sun X, Ji T, Huang H, Yang Y, Zhang H, Tahir HAS, Wu L, Wu H, Gao X (2017). FvSet2 regulates fungal growth, pathogenicity, and secondary metabolism in *Fusarium verticillioides*. Fungal Genet Biol.

[CR34] Guo M, Guo W, Chen Y, Dong S, Zhang X, Zhang H, Song W, Wang W, Wang Q, Lv R, Zhang Z, Wang Y, Zheng X (2010). The basic leucine zipper transcription factor Moatf1 mediates oxidative stress responses and is necessary for full virulence of the rice blast fungus *Magnaporthe oryzae*. MPMI.

[CR35] Hagiwara D, Asani Y, Yamashino T, Mizuno T (2008). Characterization of bZIP-type transcription factor AtfA with reference to stress responses of conidia of *Aspergillus nidulans*. Biosci Biotechnol Biochem.

[CR36] Hagiwara D, Asano Y, Marui J, Yoshimi A, Mizuno T, Abe K (2009). Transcriptional profiling for *Aspergillus nidulans* HogA MAPK signaling pathway in response to fludioxonil and osmotic stress. Fungal Genet Biol.

[CR37] Hagiwara D, Suzuki S, Kamei K, Gonoi T, Kawamoto S (2014). The role of AtfA and HOG MAPK pathway in stress tolerance in conidia of *Aspergillus fumigatus*. Fungal Genet Biol.

[CR38] Hagiwara D, Takahashi H, Kusuya Y, Kawamoto S, Kamei K, Gonoi T (2016). Comparative transcriptome analysis revealing dormant conidia and germination associated genes in *Aspergillus* species: an essential role for AtfA in conidial dormancy. BMC Genomics.

[CR39] Han Z, Tangni EK, Huybrechts B, Munaut F, Scauflaire J, Wu A, Callebaut A (2014). Screening survey of co-production of fusaric acid, fusarin C, and fumonisin B1, B2 and B3 by *Fusarium* strains grown in maize grains. Mycotoxin Res.

[CR40] Herrera ML, Vallor AC, Gelford JA, Patterson TF, Wickes BL (2009). Strain-dependent variation in 18S ribosomal DNA copy numbers in *Aspergillus fumigatus*. J Clin Microbiol.

[CR41] Hong SY, Roze LV, Wee J, Linz JE (2013). Evidence that a transcription factor regulatory network coordinates oxidative stress response and secondary metabolism in aspergilli. MicrobiologyOpen.

[CR42] Hornero-Mendez D, Limón MC, Avalos J (2018). HPLC analysis of carotenoids in neurosporaxanthin-producing fungi. In: eds. Carlos Barreiro and José-Luis Barredo (eds) Microbial carotenoids: methods and protocols. Methods Mol Biol..

[CR43] Jiang C, Zhang S, Zhang Q, Tao Y, Wang C, Xu JR (2015). *FgSKN7* and *FgATF1* have overlapping functions in ascosporogenesis, pathogenesis and stress responses in *Fusarium graminearum*. Environ Microbiol.

[CR44] Johnson ET, Proctor RH, Dunlap CA, Busman M (2018). Reducing production of fumonisin mycotoxins in *Fusarium verticillioides* by RNA interference. Mycotoxin Res.

[CR45] Jones DT, Taylor WR, Thornton JM (1992). The rapid generation of mutation data matrices from protein sequences. CABIOS.

[CR46] Kamangar F, Chow WH, Abnet CC, Dawsey SM (2009). Environmental causes of esophageal cancer. Gastroenterol Clin North Am.

[CR47] Kigen G, Busakhala N, Kamuren Z, Rono H, Kimalat W, Njiru E (2017). Factors associated with the high prevalence of oesophageal cancer in Western Kenya: a review. Infect Agent Cancer.

[CR48] Kohut G, Ádám LA, Fazekas B, Hornok L (2009). N-starvation stress induced *FUM* gene expression and fumonisin production is mediated via the HOG-type MAPK pathway in *Fusarium proliferatum*. Int J Food Microbiol.

[CR49] Kohut G, Oláh B, Adám AL, García-Martínez J, Hornok L (2010). Adenylyl cyclase regulates heavy metal sensitivity, bikaverin production and plant tissue colonization in *Fusarium proliferatum*. J Basic Microbiol.

[CR50] Kouzi, S. A., Wright, N. J. D., Dirks-Naylor, A., and Uddin, M. N (2018) Fumonisins: effects on human and animal health and mechanisms of toxicity. EC Pharmacol. Toxicol 6:187–208.

[CR51] Kozlowski LP (2016). IPC–isoelectric point calculator. Biol Direct.

[CR52] Krifka S, Spagnuolo G, Schmalz G, Schweikl H (2013). A review of adaptive mechanisms in cell responses towards oxidative stress caused by dental resin monomers. Biomaterials.

[CR53] Kumar A, Srikanta AH, Muthukumar SP, Sukumaran UK, Govindaswamy V (2011). Antioxidant and lipid peroxidation activities in rats fed with *Aspergillus carbonarius* carotenoid. Food Chem Toxicol.

[CR54] Kumar S, Stecher G, Li M, Knyaz C, Tamura K (2018). MEGA X: Molecular Evolutionary Genetics Analysis across computing platforms. Mol Biol Evol.

[CR55] Kvietikova I, Wenger RH, Marti HH, Gassmann M (1995). The transcription factors ATF-1 and CREB-1 bind constitutively to the hypoxia-inducible factor-1 (HIF-1) DNA recognition site. Nucleic Acids Res.

[CR56] Lara-Rojas F, Sánchez O, Kawasaki L, Aguirre J (2011). *Aspergillus nidulans* transcription factor AtfA interacts with the MAPK SakA to regulate general stress responses, development and spore functions. Mol Microbiol.

[CR57] Lazzaro I, Busman M, Battilani P, Butchko RAE (2012). *FUM* and *BIK* gene expression contribute to describe fumonisin and bikaverin synthesis in *Fusarium verticillioides*. Int J Food Microbiol.

[CR58] Lebeau J, Petit T, Clerc P, Dufossé L, Caro Y (2019). Isolation of two novel purple naphthoquinone pigments concomitant with the bioactive red bikaverin and derivates thereof produced by *Fusarium oxysporum*. Biotechnol Prog.

[CR59] Leiter É, Park HS, Kwon NJ, Han KH, Emri T, Oláh V, Mészáros I, Dienes B, Vincze J, Csernoch L, Yu JH, Pócsi I (2016). Characterization of the *aodA*, *dnmA*, *mnSOD* and *pimA* genes in *Aspergillus nidulans*. Sci Rep.

[CR60] Leslie JF, Summerell BA (2006) The *Fusarium* Laboratory Manual. Blackwell Publishing, pp.:60–61. 10.1002/9780470278376

[CR61] Limón MC, Rodríguez-Ortiz R, Avalos J (2010). Bikaverin production and applications. Appl Microbiol Biotechnol.

[CR62] Linnemannstöns P, Schulte J, del Mar PM, Proctor RH, Avalos J, Tudzynski B (2002). The polyketide synthase gene *pks4* from *Gibberella fujikuroi* encodes a key enzyme in the biosynthesis of the red pigment bikaverin. Fungal Genet Biol.

[CR63] Linnemannstöns P, Prado MM, Fernández-Martín R, Tudzynski B, Avalos J (2002). A carotenoid biosynthesis gene cluster in *Fusarium fujikuroi*: the genes *carB* and *carRA*. Mol Genet Genomics.

[CR64] Liu X, Fan L, Yin S, Chen H, Hu H (2019). Molecular mechanisms of fumonisin B1-induced toxicities and its applications in the mechanism-based interventions. Toxicon.

[CR65] Loeken MR (1993). Effects of mutation of the CREB binding site of the somatostatin promoter on cyclic AMP responsiveness in CV-1 cells. Gene Expr.

[CR66] Logrieco A, Mulé G, Moretti A, Bottalico A (2002). Toxigenic *Fusarium* species and mycotoxins associated with maize ear rot in Europe. Eur J Plant Pathol.

[CR67] Lumsangkul C, Chiang HI, Lo NW, Fan YK, Ju JC (2019). Developmental toxicity of mycotoxin Fumonisin B_1_ in animal embryogenesis: an overview. Toxins (Basel).

[CR68] Majumdar R, Rajasekaran K, Cary JW (2017). RNA interference (RNAi) as a potential tool for control of mycotoxin contamination in crop plants: concepts and considerations. Front Plant Sci.

[CR69] McDonald T, Brown D, Keller NP, Hammond TM (2005). RNA silencing of mycotoxin production in *Aspergillus* and *Fusarium* species. Mol Plant Microbe Interact.

[CR70] McGinnis S, Madden TL (2004) BLAST: at the core of a powerful and diverse set of sequence analysis tools. Nucleic Acids Res 32:W20–W25. 10.1093/nar/gkh435 10.1093/nar/gkh435 PMC441573 15215342

[CR71] Messeguer X, Escudero R, Farré D, Núñez O, Martínez J, Albà MM (2002). PROMO: detection of known transcription regulatory elements using species-tailored searches. Bioinformatics.

[CR72] Miskei M, Karányi Z, Pócsi I (2009). Annotation of stress-response proteins in the aspergilli. Fungal Genet Biol.

[CR73] Müller S, Dekant W, Mally A (2012). Fumonisin B1 and the kidney: modes of action for renal tumor formation by fumonisin B1 in rodents. Food Chem Toxicol.

[CR74] Nagygyörgy ED, Kovács B, Leiter E, Miskei M, Pócsi I, Hornok L, Ádám AL (2014). Toxicity of abiotic stressors to *Fusarium* species: differences in hydrogen peroxide and fungicide tolerance. Acta Microbiol Immunol Hung.

[CR75] Nair MG (2017). Fumonisins and human health. Ann. Trop. Paediatr.

[CR76] Nathues E, Joshi S, Tenberge KB, Driesch M, Oeser B, Bäumer N, Mihlan M, Tudzynski P (2004) CPTF1, a CREB-like transcription factor, is involved in the oxidative stress response in the phytopathogen *Claviceps purpurea* and modulates ROS level in its host *Secale cereale.* MPMI 383–393. 10.1094/MPMI.2004.17.4.383 10.1094/MPMI.2004.17.4.383 15077671

[CR77] Nguyen TV, Kröger C, Bönnighausen J, Schäfer W, Jörg B (2013). The ATF/CREB transcription factor Atf1 is essential for full virulence, deoxynivalenol production, and stress tolerance in the cereal pathogen *Fusarium graminearum*. MPMI.

[CR78] Niehaus EM, Schumacher J, Burkhardt I, Rabe P, Spitzer E, Münsterkötter M, Güldener U, Sieber CMK, Dickschat JS, Tudzynski B (2017) The GATA-type transcription factor Csm1 regulates conidiation and secondary metabolism in *Fusarium fujikuroi*. Front Microbiol 26;8:1175. 10.3389/fmicb.2017.01175. 10.3389/fmicb.2017.01175 PMC5483468 28694801

[CR79] Orosz E, Antal K, Gazdag Z, Szabó Z, Han KH, Yu JH, Pócsi I, Emri T (2017). Transcriptome-based modeling reveals that oxidative stress induces modulation of the AtfA-dependent signaling networks in *Aspergillus nidulans*. Int J Genomics.

[CR80] Orosz E, van de Wiele N, Emri T, Zhou M, Robert V, de Vries RP, Pócsi I (2018) Fungal Stress Database (FSD) - a repository of fungal stress physiological data. Database (Oxford) 2018:bay009. 10.1093/database/bay009 10.1093/database/bay009 PMC5810435 29688353

[CR81] Pareek M, Rajam MV (2017). RNAi-mediated silencing of MAP kinase signalling genes (*Fmk1*, *Hog1*, and *Pbs2*) in *Fusarium oxysporum* reduces pathogenesis on tomato plants. Fungal Biol.

[CR82] Pfannmüller A, Leufken J, Studt L, Michielse CB, Sieber CMK, Güldener U, Hawat S, Hippler M, Fufezan C, Tudzynski B (2017) Comparative transcriptome and proteome analysis reveals a global impact of the nitrogen regulators AreA and AreB on secondary metabolism in *Fusarium fujikuroi*. PLoS One 25;12(4):e0176194. 10.1371/journal.pone.0176194 10.1371/journal.pone.0176194 PMC5404775 28441411

[CR83] Picot A, Barreau C, Pinson-Gadais L, Caron D, Lannou C, Richard-Forget F (2010). Factors of the *Fusarium verticillioides*-maize environment modulating fumonisin production. Crit Rev Microbiol.

[CR84] Pócsi I, Miskei M, Karányi Z, Emri T, Ayoubi P, Pusztahelyi T, Balla G, Prade RA (2005). Comparison of gene expression signatures of diamide, H_2_O_2_ and menadione exposed *Aspergillus nidulans* cultures--linking genome-wide transcriptional changes to cellular physiology. BMC Genomics.

[CR85] Ponce-García N, Serna-Saldivar SO, Garcia-Lara S (2018). Fumonisins and their analogues in contaminated corn and its processed foods – a review. Food Addit Contam Part A Chem Anal Control Expo Risk Assess..

[CR86] Prado-Cabrero A, Estrada AF, Al-Babili S, Avalos J (2007). Identification and biochemical characterization of a novel carotenoid oxygenase: elucidation of the cleavage step in the *Fusarium* carotenoid pathway. Mol Microbiol.

[CR87] Prado-Cabrero A, Schaub P, Díaz-Sánchez V, Estrada AF, Al-Babili S, Avalos J (2009). Deviation of the neurosporaxanthin pathway towards beta-carotene biosynthesis in *Fusarium fujikuroi* by a point mutation in the phytoene desaturase gene. FEBS J.

[CR88] Pusztahelyi T, Klement E, Szajli E, Klem J, Miskei M, Karányi Z, Emri T, Kovács S, Orosz G, Kovács KL, Medzihradszky KF, Prade RA, Pócsi I (2011). Comparison of transcriptional and translational changes caused by long-term menadione exposure in *Aspergillus nidulans*. Fungal Genet Biol.

[CR89] Qi X, Guo L, Yang L, Huang J (2013). Foatf1, a bZIP transcription factor of *Fusarium oxysporum* f. sp. *cubense* is involved in pathogenesis by regulating the oxidative stress responses of Cavendish banana *(Musa* spp.). Physiol Mol Plant P.

[CR90] Ray WC (2005). MAVL/StickWRLD for protein: visualizing protein sequence families to detect non-consensus features. Nucleic Acids Res.

[CR91] Rodrigues-Pousada C, Menezes RA, Pimentel C (2010). The Yap family and its role in stress response. Yeast.

[CR92] Rösler SM, Kramer K, Finkemeier I, Humpf HU, Tudzynski B (2016). The SAGA complex in the rice pathogen *Fusarium fujikuroi*: structure and functional characterization. Mol Microbiol.

[CR93] Roze LV, Chanda A, Wee J, Awad D, Linz JE (2011). Stress-related transcription factor AtfB integrates secondary metabolism with oxidative stress response in aspergilli. J Biol Chem.

[CR94] Sagaram U, Shaw BD, Shim WB (2007). *Fusarium verticillioides GAP1*, a gene encoding a putative glycolipid-anchored surface protein, participates in conidiation and cell wall structure but not virulence. Microbiology.

[CR95] Sakamoto K, Arima TH, Iwashita K, Yamada O, Gomi K, Akita O (2008). *Aspergillus oryzae atfB* encodes a transcription factor required for stress tolerance in conidia. Fungal Genet Biol.

[CR96] Sakamoto K, Iwashita K, Yamada O, Kobayashi K, Mizuno A, Akita O, Mikami S, Shimoi H, Gomi K (2009). *Aspergillus oryzae* atfA controls conidial germination and sress tolerance. Fungal Genet Biol.

[CR97] Sansó M, Gogol M, Ayté J, Seidel C, Hidalgo E (2008). Transcription factor Pcr1 and Atf1 have distinct roles in stress- and Sty1-dependent gene regulation. Eukaryot Cell.

[CR98] Sansó M, Vargas-Pérez I, García P, Ayté J, Hidalgo E (2011). Nuclear roles and regulation of chromatin structure by the stress-dependent MAP kinase Sty1 of *Schizosaccharomyces pombe*. Mol Microbiol.

[CR99] Segorbe D, Di Pietro A, Pérez-Nadales E, Turrà D (2017). Three *Fusarium oxysporum* mitogen-activated protein kinases (MAPKs) have distinct and complementary roles in stress adaptation and cross-kingdom pathogenicity. Mol Plant Pathol.

[CR100] Seong KH, Maekawa T, Ishii S (2012). Inheritance and memory of stress-induced epigenome change: roles played by the ATF-2 family of transcription factors. Genes Cells.

[CR101] Setnikar I, Senin P, Rovati LC (2005). Antiatherosclerotic efficacy of policosanol, red yeast rice extract and astaxanthin in the rabbit. Arzneimittelforschung..

[CR102] Shim WB, Sagaram US, Choi YE, So J, Wilkinson HH, Lee YW (2006). *FSR1* is essential for virulence and female fertility in *Fusarium verticillioides* and *F. graminearum*. Mol Plant Microbe Interact.

[CR103] Shiozaki K, Russel P (1996). Conjugation, meiosis, and the osmotic stress response are regulated by Spc1 kinase through Atf1 transcription factor in fission yeast. Gene Dev.

[CR104] Solovyev V, Kosarev P, Seledsov I, Vorobyev D (2006). Automatic annotation of eukaryotic genes, pseudogenes and promoters. Genome Biol.

[CR105] Son SW, Kim HY, Choi GJ, Lim HK, Jang KS, Lee SO, Lee S, Sung ND, Kim JC (2008). Bikaverin and fusaric acid from *Fusarium oxysporum* show antioomycete activity against Phytophthora infestans. J Appl Microbiol.

[CR106] Sondergaard TE, Fredborg M, Oppenhagen Christensen AM, Damsgaard SK, Kramer NF, Giese H, Sørensen JL (2016). Fast screening of antibacterial compounds from Fusaria. Toxins (Basel).

[CR107] Studt L, Humpf HU, Tudzynski B (2013). Signaling governed by G proteins and cAMP is crucial for growth, secondary metabolism and sexual development in *Fusarium fujikuroi*. PLoS One.

[CR108] Takeda T, Toda T, Kominami K, Kohnosu A, Yanagida M, Jones N (1995). *Schizosaccharomyces pombe* atf1+ encodes a transcription factor required for sexual development and entry into stationary phase. EMBO J.

[CR109] Teichert S, Wottawa M, Schönig B, Tudzynski B (2006). Role of the *Fusarium fujikuroi* TOR kinase in nitrogen regulation and secondary metabolism. Eukaryot Cell.

[CR110] Temme N, Oeser B, Massaroli M, Heller J, Simon A, Collado IG, Viaud M, Tudzynski P (2012). BcAtf1, a global regulator, controls various differentiation processes and phytotoxin production in *Botrytis cinerea*. Mol Plant Pathol.

[CR111] Thomas AB, Shetane TD, Singha RG, Nanda RK, Poddar SS, Shirsat A (2017). Employing central composite design for evaluation of biomass production by *Fusarium venenatum*: In vivo antioxidant and antihyperlipidemic properties. Appl Biochem Biotechnol.

[CR112] Vlahopoulos SA, Logotheti S, Mikas D, Giarika A, Gorgoulis V, Zoumpourlis V (2008). The role of ATF-2 in oncogenesis. Bioessays.

[CR113] Wagner D, Schmeinck A, Mos M, Morozov IY, Caddick MX, Tudzynski B (2010). The bZIP transcription factor MeaB mediates nitrogen metabolite repression at specific loci. Eukaryot Cell.

[CR114] Wee J, Hong SY, Roze LV, Day DM, Chanda A, Linz JE (2017). The fungal bZIP transcription factor AtfB controls virulence-associated processes in *Aspergillus parasiticus*. Toxins (Basel).

[CR115] Wiemann P, Willmann A, Straeten M, Kleigrewe K, Beyer M, Humpf HU, Tudzynski B (2009). Biosynthesis of the red pigment bikaverin in *Fusarium fujikuroi*: genes, their function and regulation. Mol Microbiol..

[CR116] Wiemann P, Brown DW, Kleigrewe K, Bok JW, Keller NP, Humpf HU, Tudzynski B (2010). FfVel1 and FfLae1, components of a velvet-like complex in *Fusarium fujikuroi*, affect differentiation, secondary metabolism and virulence. Mol Microbiol.

[CR117] Wolf T, Shelest V, Nath N, Shelest E, (2016) CASSIS and SMIPS: promoter-based prediction of secondary metabolite gene clusters in eukaryotic genomes. Bioinformatics 32(8):1138–1143. 10.1093/bioinformatics/btv713 10.1093/bioinformatics/btv713 PMC4824125 26656005

[CR118] Woloshuk CP, Shim WB (2013). Aflatoxins, fumonisins, and trichothecenes: a convergence of knowledge. FEMS Microbiol Rev.

[CR119] Wu F, Groopman JD, Pestka JJ (2014). Public health impacts of foodborne mycotoxins. Annu Rev Food Sci Technol.

[CR120] Yu JH, Hamari Z, Han KH, Seo JA, Reyes-Dominguez Y, Scazzocchi C (2004). Double-joint PCR: a PCR-based molecular tool for gene manipulations in filamentous fungi. Fungal Genet Biol.

[CR121] Yu CP, Lin JJ, Li WH (2016). Positional distribution of transcription factor binding sites in *Arabidopsis thaliana*. Sci Rep.

[CR122] Zhan J, Burns AM, Liu MX, Faeth SH, Gunatilaka AA (2007). Search for cell motility and angiogenesis inhibitors with potential anticancer activity: beauvericin and other constituents of two endophytic strains of *Fusarium oxysporum*. J Nat Prod.

[CR123] Zhang Y, Choi YE, Zou X, Xu JR (2011). The *FvMK1* mitogen-activated protein kinase gene regulates conidiation, pathogenesis, and fumonisin production in *Fusarium verticillioides*. Fungal Genet Biol.

