# Supplementary material for: FvatfA regulates growth, stress tolerance as well as mycotoxin and pigment productions in Fusarium verticillioides
Source: Appl Microbiol Biotechnol. 2020 Jul 27;104(18):7879–99. doi: 10.1007/s00253-020-10717-6 (PMC7447684; doi:10.1007/s00253-020-10717-6)
Supplement: Supplementary file 1 — (PDF 1.33 mb) [file 253_2020_10717_MOESM1_ESM.pdf]

Applied Microbiology and Biotechnology

Supplementary Material

FvatfA regulates growth, stress tolerance as well as mycotoxin and pigment productions in *Fusarium verticillioides*

Zsuzsa Szabó[1,2], Klaudia Pákozdi[1,3], Katalin Murvai[1], Tünde Pusztahelyi[4], Ádám Kecskeméti[5], Attila Gáspár[5], Antonio F. Logrieco[6], Tamás Emri[1], Attila L. Ádám[7], Éva Leiter[1], László Hornok[8] and István Pócsi[1]

Affiliations

[1] Department of Molecular Biotechnology and Microbiology, Institute of Biotechnology, Faculty of Science and Technology, University of Debrecen, Debrecen, Hungary

[2] Doctoral School of Biological Sciences, Faculty of Agricultural and Environmental Sciences, Szent István University, Gödöllő, Hungary

[3] Doctoral School of Nutrition and Food Sciences, Faculty of Medicine, University of Debrecen, Debrecen, Hungary

[4] Central Laboratory of Agricultural and Food Products, Faculty of Agricultural and Food Sciences and Environmental Management, University of Debrecen, Debrecen, Hungary

[5] Department of Inorganic and Analytical Chemistry, Faculty of Science and Technology, University of Debrecen, Debrecen, Hungary

[6] Institute of Sciences of Food Production (ISPA-CNR), Bari, Italy

[7] Plant Protection Institute, Centre for Agricultural Research, Budapest, Hungary

[8] Faculty of Agricultural and Environmental Sciences, Szent István University, Gödöllő, Hungary

Corresponding author

*I. Pócsi, e-mail: pocsi.istvan@science.unideb.hu, telephone: +36-52-512900 ext. 62337, ORCID ID: 0000-0003-2692-6453

Supplementary Tables

Supplementary Table S1. Homologies between FvAtfA orthologous proteins identified in various fungal species.

Species	Expect value (blastp to FVEG_02866)	Name	NCBI Accession number
Fusarium verticillioides	0.0	FVEG_02866 hypothetical protein (FvAtfA)	XP_018746682.1
Fusarium oxysporum f. sp. cubense	0.0	FoAtf1 (Qi et al. 2013)	TVY66251.1
Fusarium graminearum	0.0	FgAtf1 (Nguyen et al. 2013; Jiang et al. 2015)	XP_011319081.1
Aspergillus nidulans	3e-96	AtfA (Hagiwara et al. 2009; Balázs et al. 2010)	ACJ61780.1
Aspergillus oryzae	3e-96	AtfA (Sakamoto et al. 2009)	XP_001819834.1
Aspergillus fumigatus	2e-102	AtfA (Hagiwara et al. 2014)	XP_754486.2
Schizosaccharomyces pombe	1e-33	Atf1 (Takeda et al. 1995)	BAA12194.1
Homo sapiens	4e-21	ATF-2 (Venter et al. 2001)	EAX11109.1
Claviceps purpurea	4e-149	CPTF1 (Nathues et al. 2004)	CCE33955.1
Magnaporthe oryzae	8e-122	Moatf1 (Guo et al. 2010)	XP_003715195.1
Botrytis cinerea	6e-32	BcAtf1 (Temme et al. 2012)	CCD34747.1
Penicillium marneffei	6e-87	AtfA (Nimmanee et al. 2014)	XP_002143956.1
Neurospora crassa	9e-147	ATF-1 (Colot et al. 2006)	XP_011394512.1

Supplementary Table S2. Oligonucleotides used in this study

**Name**	**Sequence (5’-3’)**	**Purpose**
**Primers used to generate the gene deletion and complemented strains**		
FvatfAupfwd	TTCATTCTGGGTTCCTTCTATGGC	5’-flanking region of *FvatfA*
FvatfAdownrev	TCCTCCTTGTCGATGTTCTTTTAG	3’-flanking region of *FvatfA*
FvatfAupkimrev	TCACTGGCCGTCGTTTTACAAAGATCCGCCGGCCTGAACCGCGAA	5’ *FvatfA* with *hph* tail
FvatfAdownkimfwd	CATGGTCATAGCTGTTTCCTGACTTCATTTCGCGTTTGAGCTGTA	3’ *FvatfA* with *hph* tail
FvatfAnestedfwd	ACAGTGCCTACAGTGTGAAGCAGG	5’ nested of *FvatfA*
FvatfAnestedrev	GGTTCCATTTGTTGTCGGTGCCTT	3’ nested of *FvatfA*
M13F	TTGTAAAACGACGGCCAGTGA	5’ *hph*/*gen* marker gener
M13R	CAGGAAACAGCTATGACCATG	3’ *hph*/*gen* marker gene
FvatfAcompkimfwd	CATGGTCATAGCTGTTTCCTGACAGTGCCTACAGTGTGAAGCAGG	5’ *FvatfA* with *gen* tail
FvatfAcomprev	TCTCAATCGCAGCTTGTGTTGCTT	3’ *FvatfA* flaking region outside the terminator region
**Primers used in copy number determinations**		
FVEG2866shortfwd	CTTGAGGACGGGTTTGACAC	forward primer of the *FvatfA* gene
FVEG2866shortrev	CGCTTCGGACTTATTGGTTG	reverse primer of the *FvatfA* gene
FVEG11192shortfwd	CAAGCACCACCAGACCTATGT	forward primer of the *FvmnSOD* gene
FVEG11192shortrev	CTCCCAAGCGTCAATACCG	reverse primer of the *FvmnSOD* gene
**Primers used in qRT-PCR experiments**		
carRAF	AGCAAACCGACCTTTCAC	forward primer of the *carRA* gene
carRAR	CCATCCCACAAGACAATG	reverse primer of the *carRA* gene
carBF	CTTCTCCTCCCTCGCTTCTTC	forward primer of the *carB* gene
carBR	CTCGCCAGACTCAAAATGCTAG	reverse primer of the *carB* gene
carTF	ATGAGTGGATTGCTGATGTG	forward primer of the *carT* gene
carTR	GGGGTTGAAGAGTTGAAGG	reverse primer of the *carT* gene
fum1F	ACAGCCCAAGCAGTAAGC	forward primer of the *fum1* gene
fum1R	CACCCGACACACGATATG	reverse primer of the *fum1* gene
fum8F	AGCATCCAACAGAAATACGC	forward primer of the *fum8* gene
fum8R	GCCTCTCTCATTGAAACGAC	reverse primer of the *fum8* gene
fum21F	CCATTACAAGCCATTCCAC	forward primer of the *fum21* gene
fum21R	ACAAGCCACGATTTAGACG	reverse primer of the *fum21* gene
bik1F	GAGCACACACCAACCATTC	forward primer of the *bik1* gene
bik1R	GACTGAGCCGAGATACAAGC	reverse primer of the *bik1* gene
tef1F	GAGCGTGAGCGTGGTATC	forward primer of the *tef1* reference gene
tef1R	CGAGGGTGTAGGCAAGAAG	reverse primer of the *tef1* reference gene
tub2F	CGGTCAGTGCGGTAACCAA	forward primer of the *tub2* reference gene
tub2R	GGCTCGGGGAACATACTTGT	reverse primer of the *tub2* reference gene
cyp2F	CTACCCCGCCATAACTGCTT	forward primer of the *cyp2* reference gene
cyp2R	AGAGCACGGAAGTTCTCAGC	reverse primer of the *cyp2* reference gene

Supplementary Table S3. Names and functions of *Fusarium verticillioides* genes used in qRT-PCR experiments

**Name**	**Accession number**	**Function**	**Reference**
fum1	FVEG_00316	polyketide synthase essential for fumonisin production	Butchko et al. 2012
fum8	FVEG_14635	α-oxoamine synthase	Lazzaro et al. 2012
fum21	FVEG_14633	transcription factor with Zn(II)2Cys6 DNA-binding domain	Butchko et al. 2012
carRA	FVEG_10718	bifunctional enzyme with phytoene synthase and carotene cyclase activities	Ádám et al. 2011
carB	FVEG_10717	carotene desaturase	Ádám et al. 2011
carT	FVEG_09251	carotene-cleaving oxygenase	Ádám et al. 2011
bik1	FVEG_03379	polyketide synthase essential for bikaverin production	Lazzaro et al. 2012
tef1	FVEG_02381	translation elongation factor 1-α	Brown et al. 2007
tub2	FVEG_04081	β-tubulin	Lazzaro et al. 2012
cyp2	FVEG_00403	putative cyclophilin	Johnson et al. 2018

Supplementary Table S4. Results of the *in silico* promoter analyses for putative ATF/CREB transcription factor binding motifs

Transcription factor	ID	Species
ATF	[T00049]	*Saccharomyces cerevisiae*
ATF	[T00051]	*Homo sapiens*
ATF-1	[T00968]	*Homo sapiens*
ATF-2	[T00167]	*Homo sapiens*
ATF3	[T01313]	*Homo sapiens*
CREB	[T00163]	*Homo sapiens*
CREB	[T00164]	*Rattus norvegicus*
CREB	[T00989]	*Mus musculus*
CRE-BP2	[T01017]	*Mus musculus*

Gene	Transcription factor	ID	Sequence	Position	Dissimilarity (%)
fum1	ATF3	[T01313]	TGACTCAA	- 464 - -457	7.45
			TGACATCT	-360 - -353	7.45
fum8	ATF3	[T01313]	TGACTTGT	-162 - -155	4.93
			TGACTACC	107-114	12.39
			TGACATCC	125-132	8.66
			ATCCGTCA	209-216	7.45
			CCTAGTCA	359-369	13.59
			TGACTCGA	384-391	8.66
			GAAAGTCA	452-459	6.14
			TTGTGTCA	486-493	11.18
fum21	ATF3	[T01313]	ATTAGTCA	63-70	11.18
			TATCGTCA	205-212	8.66
			TGACAGAT	591-598	14.91
			TGACTACT	742-749	11.18
			CCAAGTCA	780-787	6.14
			TCCCGTCA	791-798	8.66
			CATAGTCA	807-814	13.59
			TGACGCCT	949-956	3.73
			TGACTGTA	1064-1071	12.39

Gene	Transcription factor	ID	Sequence	Position	Dissimilarity (%)
carRA	ATF	[T00049]	CGTGACGTCA	254-263	0.48
			TGACGTCAAC	256-265	0.24
	ATF	[T00051]	GTGACGTCA	255-263	0.31
			TGACGTCAA	256-264	0.54
	ATF-2	[T00167]	GTGACGTCA	255-263	0.00
			TGACGTCAA	256-264	0.05
	ATF-1	[T00968]	CGTGACGTCAA	254-264	4.58
			GTGACGTCAAC	255-265	2.83
	ATF3	[T01313]	GTATGTCA	-76 - -83	8.66
			ATTTGTCA	114-121	14.91
			ACGCGTCA	121-128	4.93
			CAATGTCA	160-167	9.86
			TGACGTCA	256-263	0.00
			TGACAGAA	285-292	14.91
			TGACTCTG	477-484	9.86
	CREB	[T00163]	GTGACGTCA	255-263	0.02
			TGACGTCAA	256-264	0.02
	CREB	[T00164]	GTGACGTCA	255-263	0.00
			TGACGTCAA	256-264	0.02
	CRE-BP2	[T01017]	GTGACGTCA	255-263	0.00
			TGACGTCAA	256-264	0.47
			ATGCCGTCA	508-516	13.99
			TGCCGTCAC	509-517	13.52
	CREB	[T00989]	CGTGACGTCA	254-263	0.00
			TGACGTCAAC	256-265	0.48
			TGCCGTCACG	509-518	14.72
carB	ATF	[T00049]	GAAGACGTCA	204-213	2.28
	ATF	[T00051]	AAGACGTCA	205-213	1.29
	ATF-1	[T00968]	GAAGACGTCAT	204-214	3.49
	ATF-2	[T00167]	AAGACGTCA	205-213	1.85
	ATF3	[T01313]	ACCCGTCA	23-30	8.66
			GATCGTCA	33-40	9.86
			AGACGTCA	206-213	0.00
			TGACTCTA	215-222	8.66
			TGACTGAG	267-274	12.39
	CREB	[T00989]	GAAGACGTCA	204-213	6.12
	CREB	[T00163]	AAGACGTCA	205-213	0.86
	CREB	[T00164]	AAGACGTCA	205-213	3.17
	CRE-BP2	[T01017]	AAGACGTCA	205-213	13.99
			AGACGTCAT	206-214	13.99

Gene	Transcription factor	ID	Sequence	Position	Dissimilarity (%)
carT	ATF3	[T01313]	TTCCGTCA	-56 - -63	7.45
			TCGAGTCA	22-29	8.66
			ACTCGTCA	180-187	8.66
			TAGGGTCA	212-219	12.39
			AAGAGTCA	395-402	8.66
			AGTCGTCA	418-425	7.45
			TCTCGTCA	482-489	8.66
			TGTCGTCA	580-587	7.45
			TAGCGTCA	814-821	4.93
			TGACTTTA	823-830	4.93
			TGACGACA	959-966	7.45
	CRE-BP2	[T01017]	TTGTCGTCA	579-587	13.99
			TGTCGTCAG	580-588	13.99
			ATGACGACA	958-966	13.99
			TGACGACAG	959-967	13.99

bik1	ATF3	[T01313]	TGACTCGA	-67 - -60	8.66
			TGACCCTG	71-78	13.59
			AAGGGTCA	158-165	12.39
	CREB	[T00164]	CAAGGAGAG	-172 - -164	10.36
			CTGACCCTG	70-78	14.90

* - Positions are counted starting from the TSS of the DNA sequence of genes selected for promoter analyses (Supplementary Figure S6).

Supplementary Table S5. Results of One-Way ANOVA analysis.

Studied property		Strains				One-Way ANOVA	Tukey post hoc test			Number of biological replicates with each strain
		FGSC 7600 (A)	*ΔFvatfA* (B)	*FvatfA* 'C (H7) (C)	*FvatfA* 'C (H9) (D)	p - value (p <)	adj. p-value			
							B vs. A	B vs. C	B vs. D	
**Vegetative growth (colony diameter. mm)**	**Czapek-Dox agar plates**	79.50±0.60	70.13±1.15	77.75±1.39	77.92±1.51	2.20E-16	0.00E+00	0.00E+00	0.00E+00	3
	**Potato Dextrose agar plates**	81.83±0.39	63.71±1.12	82.00±0.43	82.08±0.29	2.20E-16	0.00E+00	0.00E+00	0.00E+00	3
**Invasive growth (colony diameter. mm)**	**72 h tomato**	7.92±2.07	2.25±0.87	6.58±1.38	7.42±1.98	1.19E-10	0.00E+00	4.00E-07	0.00E+00	3
	**72 h Czapek-Dox agar plate**	36.67±0.58	30.33±1.15	37.33±0.58	38.67±1.53	4.42E-05	3.32E-04	1.63E-04	4.57E-05	3
	**96 h tomato**	12.75±4.16	3.08±0.79	10.33±3.73	11.50±3.80	3.42E-08	1.00E-07	2.68E-05	1.60E-06	3
	**96 h Czapek-Dox agar plate**	53.33±1.53	43.67±0.58	52.00±2.65	51.67±0.58	2.68E-04	3.22E-04	8.94E-04	1.17E-03	3
**Spore production ($x \times 10^7/cm^2$)**		6.21±0.29	3.98±0.36	5.97±0.14	6.24±0.41	5.85E-05	1.18E-04	2.65E-04	1.08E-04	3
**Spore size (µm)**	**Arc lenght**	10.43±0.23	8.07±0.13	8.95±0.41	9.02±0.90	3.25E-03	2.07E-03	2.22E-01	1.78E-01	3
	**Spore diameter**	4.85±0.66	4.94±0.18	3.93±0.51	4.19±0.51	9.64E-02	9.96E-01	1.36E-01	3.16E-01	3
**Spore viability (number of colonies)**	**25 °C**	86.67±10.67	81.67±7.42	71.78±11.72	87.44±11.63	1.25E-02	7.45E-01	2.10E-01	6.52E-01	3
	**42 °C**	74.67±11.62	71.44±11.96	68.33±7.38	71.33±8.36	6.16E-01	9.02E-01	9.11E-01	1.00E+00	3
	**45 °C**	46.67±6.63	39.89±5.75	45.56±7.38	49.33±5.00	2.30E-02	1.20E-01	2.40E-01	1.55E-02	3
	**4 °C**	25.00±8.83	24.11±6.60	32.00±4.61	30.11±6.11	4.70E-02	9.92E-01	8.02E-02	2.50E-01	3
**Abiotic stress tolerance (relative growth)**	**H_2O_2**	74.59±1.23	40.04±0.94	68.23±5.96	66.87±7.34	9.84E-05	9.88E-05	4.21E-04	5.93E-04	3
	***t*BOOH**	54.58±0.69	47.83±1.75	50.55±2.83	50.67±2.55	2.87E-02	1.93E-02	4.46E-01	4.13E-01	3
	**MSB**	6.39±2.70	39.76±7.22	59.14±4.02	58.57±3.45	1.80E-03	2.87E-03	4.22E-03	5.07E-03	3
	**CR**	52.91±2.90	40.22±2.27	48.51±3.27	49.60±2.57	3.02E-03	2.31E-03	2.69E-02	1.40E-02	3
**Fumonisin production (µg/g DCM)**	**FB1**	37.27±4.89	0.00±0.00	37.34±11.17	28.44±8.81	4.83E-06	4.00E-06	1.60E-04	1.04E-04	5
	**FB2**	8.78±1.92	0.00±0.00	5.62±1.86	6.32±1.01	3.58E-07	2.00E-07	6.37E-05	1.65E-05	5
**Carotenoid production (µg/g DCM)**		19.44±2.68	0.47±0.03	24.46±8.69	27.05±4.87	9.08E-04	8.60E-03	2.02E-03	1.04E-03	3
**Bikaverin production (mg/g DCM)**	**day 5**	4.26±1.12	41.37±13.32	9.57±4.06	9.50±4.24	4.87E-12	0.00E+00	0.00E+00	0.00E+00	3
	**day 7**	7.18±5.36	52.50±17.45	14.31±5.90	14.44±7.24	2.13E-10	0.00E+00	0.00E+00	0.00E+00	3
	**day 9**	9.79±2.56	54.50±5.67	15.60±7.03	11.53±6.09	2.20E-16	0.00E+00	0.00E+00	0.00E+00	3

**Gene expression (-ΔC_T)**	***fum1***	8.10±0.88	12.10±0.31	8.50±0.26	8.86±0.05	2.93E-05	3.81E-05	8.31E-05	1.76E-04	3
	fum8	5.19±0.30	8.78±0.16	4.08±0.50	4.84±0.20	4.13E-07	3.60E-06	4.00E-07	1.70E-06	3
	fum21	6.25±0.52	6.57±0.52	4.95±0.54	5.54±0.45	1.84E-02	8.64E-01	1.93E-02	1.36E-01	3
	***bik1* (day 3)**	6.93±2.93	11.46±0.69	7.85±0.48	8.15±0.83	3.55E-02	3.23E-02	8.85E-02	1.23E-01	3
	***bik1* (day 5)**	10.65±0.80	12.94±2.59	8.51±2.28	10.00±2.98	2.07E-01	6.36E-01	1.66E-01	4.51E-01	3
	carRA	7.19±1.22	17.12±1.29	6.33±0.94	5.50±1.73	1.54E-05	7.46E-05	4.06E-05	2.32E-05	3
	carB	4.68±2.00	13.94±0.83	4.56±1.08	3.22±1.90	9.35E-05	3.54E-04	3.23E-04	1.25E-04	3
	carT	9.79±0.99	10.67±1.54	10.40±1.40	9.93±1.26	8.31E-01	8.43E-01	9.94E-01	8.97E-01	3

Supplementary Table S6. Two-Way ANOVA analysis of abiotic stress tolerance

Studied property		Strains								Two-Way ANOVA		
		**FGSC 7600 (A)**		***ΔFvatfA*** **(B)**		***FvatfA*** **'C (H7) (C)**		***FvatfA*** **'C (H9) (D)**		**p-value (strain)**	**p-value (treatment)**	**p-value (interaction between strain and treatment)**
		**untreated**	**treated**	**untreated**	**treated**	**untreated**	**treated**	**untreated**	**treated**			
**Abiotic stress tolerance (colony diameter. mm)**	H_2O_2	79.21±0.52	59.08±1.01	70.04±1.45	28.04±1.64	78.17±1.94	53.29±4.08	78.17±1.94	52.25±5.52	2.20E-16	2.20E-16	2.20E-16
	*t*BOOH	79.46±1.13	43.38±1.09	69.75±1.44	33.38±1.89	78.17±1.94	39.54±3.07	78.17±1.94	39.63±2.70	2.00E-16	2.00E-16	8.87E-02
	MSB	78.33±1.50	47.29±1.91	70.92±1.54	28.17±4.82	77.46±1.66	45.79±2.77	77.71±1.55	45.50±2.38	2.20E-16	2.20E-16	4.10E-15
	CR	79.38±1.00	42.00±2.46	69.88±1.23	28.13±2.07	78.17±1.94	37.88±1.69	78.17±1.94	38.79±2.77	2.20E-16	2.20E-16	1.20E-03

Studied property		Tukey post hoc test (adj. p-value)					
		**B vs. A (untreated)**	**B vs. C (untreated)**	**B vs. D (untreated)**	**B vs. A (treated)**	**B vs. C (treated)**	**B vs. D (treated)**
**Abiotic stress tolerance (colony diameter. mm)**	H_2O_2	2.00E-07	5.30E-06	5.30E-06	0.00E+00	0.00E+00	0.00E+00
	*t*BOOH	0.00E+00	0.00E+00	0.00E+00	0.00E+00	0.00E+00	0.00E+00
	MSB	0.00E+00	0.00E+00	0.00E+00	0.00E+00	0.00E+00	0.00E+00
	CR	0.00E+00	0.00E+00	0.00E+00	0.00E+00	0.00E+00	0.00E+00

Supplementary Table S7. Distribution of fumonisins FB1 and FB2 between culture supernatants and mycelia in *Fusarium verticillioides* static liquid cultures

	**FB1**		**FB2**	
**Strains**	**In supernatant (µg/g DCM)**	**In mycelium (µg/g DCM)**	**In supernatant (µg/g DCM)**	**In mycelium (µg/g DCM)**
FGSC 7600	31.02±4.71 (80.44±2.44%)	7.68±2.36 (19.56±2.44%)	5.26±0.95 (83.78±2.04%)	1.03±0.31 (16.22±2.04%)
ΔFvatfA	0.00±0.00 (0.00±0.00%)	0.00±0.00 (0.00±0.00%)	0.00±0.00 (0.00±0.00%)	0.00±0.00 (0.00±0.00%)
FvatfA ’C (H7)	26.17±5.76 (83.98±6.25%)	4.85±1.62 (16.02±6.25%)	3.63±1.76 (80.06±10.09%)	0.79±0.20 (19.94±10.09%)
FvatfA ’C (H9)	37.16±11.75 (90.78±3.62%)	3.56±1.28 (9.22±3.62%)	3.94±0.24 (84.54±3.78%)	0.72±0.18 (15.46±3.78%)

Supplementary Table S8. Results of the PCA analysis

Coordinates of the strains:

	Coordinates			
**Strain**	**PC1**	**PC2**	**PC3**	**PC4**
**FGSC 7600**	-2.96274	-3.23396	-0.40362	3.00E-16
ΔFvatfA	7.161313	-0.30343	-0.03184	1.84E-15
***FvatfA* 'C (H9)**	-2.08427	2.352623	-1.5677	-1.34E-14
***FvatfA* 'C (H9)**	-2.11431	1.184762	2.003158	1.90E-14

Importance of components:

	**PC1**	**PC2**	**PC3**	**PC4**
**Standard deviation**	4.7915	2.4145	1.48708	2.335E-15
**Proportion of Variance**	0.7406	0.1880	0.07134	0
**Cumulative Proportion**	0.7406	0.9287	1	1

**Studied property**		**PC1**[1]	**PC2**[1]	**PC3**	**PC4**
**Vegetative growth (colony diameter. mm)**	**Czapek-Dox agar plates**	-0.20758	-0.04279	-0.00465	0.434012
	**Potato Dextrose agar plates**	-0.20774	0.038742	0.013478	0.055648
**Invasive growth (colony diameter. mm)**	**72 h tomato**	-0.20622	-0.04442	0.073948	-0.09748
	**72 h Czapek-Dox agar plate**	-0.20608	-0.05582	0.055358	-0.04607
	**96 h tomato**	-0.19971	0.090416	0.128655	-0.14456
	**96 h Czapek-Dox agar plate**	-0.20797	-0.02835	-0.03227	0.328134
**Spore size (µm)**	**Arc lenght**	-0.16154	-0.259	-0.06652	0.13909
	**Spore diameter**	0.116462	-0.34304	0.034062	0.222351
**Spore viability (number of colonies)**	**25 °C**	-0.01292	-0.25843	0.523838	-0.41231
	**42 °C**	-0.01587	-0.39504	0.19545	0.023971
	**45 °C**	-0.18929	0.037402	0.27661	0.223427
	**4 °C**	-0.11983	0.338667	-0.0277	0.217727
**Spore production $(x \times 10^7/cm^2)$**		-0.20756	0.007685	0.069213	-0.03962
**Abiotic stress tolerance (relative growth)**	**H_2O_2**	-0.09997	-0.30782	-0.31408	-0.06236
	***t*BOOH**	0.207575	-0.04091	0.021349	-0.14876
	**MSB**	-0.10626	-0.24973	-0.413	-0.31792
	**CR**	-0.11249	-0.2497	-0.39555	-0.0449
**Fumonisin production (µg/g DCM)**	**FB1**	-0.20478	-0.07977	-0.0087	0.01921
	**FB2**	-0.19993	-0.11855	0.012671	-0.04186
**Bikaverin production (mg/g DCM)**	**day 5**	0.2083	0.025066	0.00881	0.009269
	**day 7**	0.208015	0.032401	0.014124	0.090683
	**day 9**	0.208101	0.005846	-0.05006	0.03274
**Carotenoid production (µg/g DCM)**		-0.19628	0.126594	0.099979	-0.10448
**Gene expression ($-\Delta C_T$)**	***fum1***	-0.20757	-0.01958	-0.06238	0.021576
	fum8	-0.19977	0.112684	-0.06622	-0.00509
	fum21	-0.13033	0.314506	-0.12283	-0.4052
	***bik1* (day 3)**	-0.20512	-0.06493	-0.06522	-0.03442
	***bik1* (day 5)**	-0.17524	0.201537	-0.16213	-0.057
	carRA	-0.20433	0.073964	0.065656	-0.06968
	carB	-0.2048	0.055817	0.092464	-0.06653
	carT	-0.16848	-0.18475	0.259818	-0.01311

[1] - Properties highlighted with blue, red and green are important component of PC1, PC2 or both, respectively. The PC1 (*i.e.* the difference between the mutant and the other strains) highly depended on: growth, invasive growth, stress tolerance, spore morphology, spore production and secondary

metabolite production. The PC2 (*i.e.* the difference between the reference and the complemented strains) highly depended on: spore viability at 4, 25 and 42 °C, spore morphology, stress tolerance (excluding *t*BOOH and 45 °C heat stress) as well as on the expression of *bik1* (day 5) and *fum21* genes.

Supplementary Figures

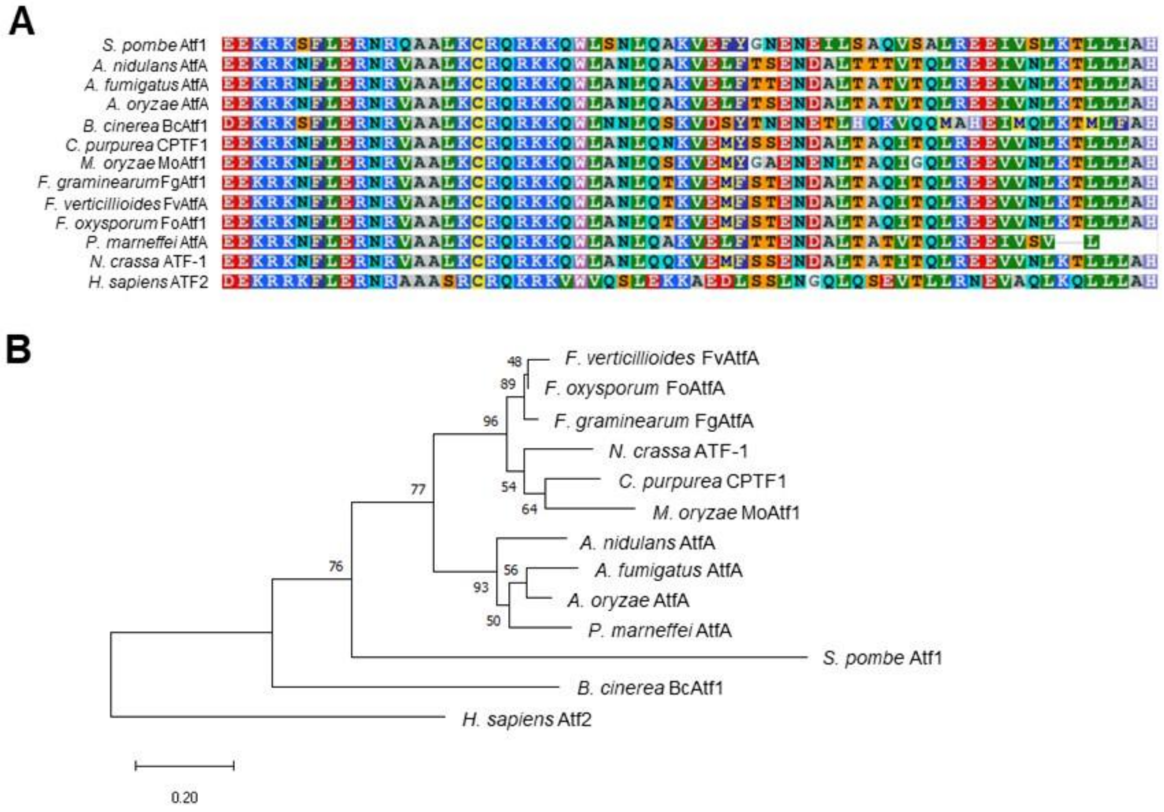

Supplementary Figure S1. Multiple sequence alignment and phylogenetic tree construction. In Part A, alignment of bZIP domains of the Atf1/AtfA ortholog transcription factors is presented. In Part B, the phylogenetic tree constructed by the Maximum Likelihood method and JTT matrix-based model is shown. Homology E expect values in comparison to *F. verticillioides* to FVEG_02866 (putatively encoding FvAtfA; NCBI blastp analysis) are summarized in Supplementary Table S1.

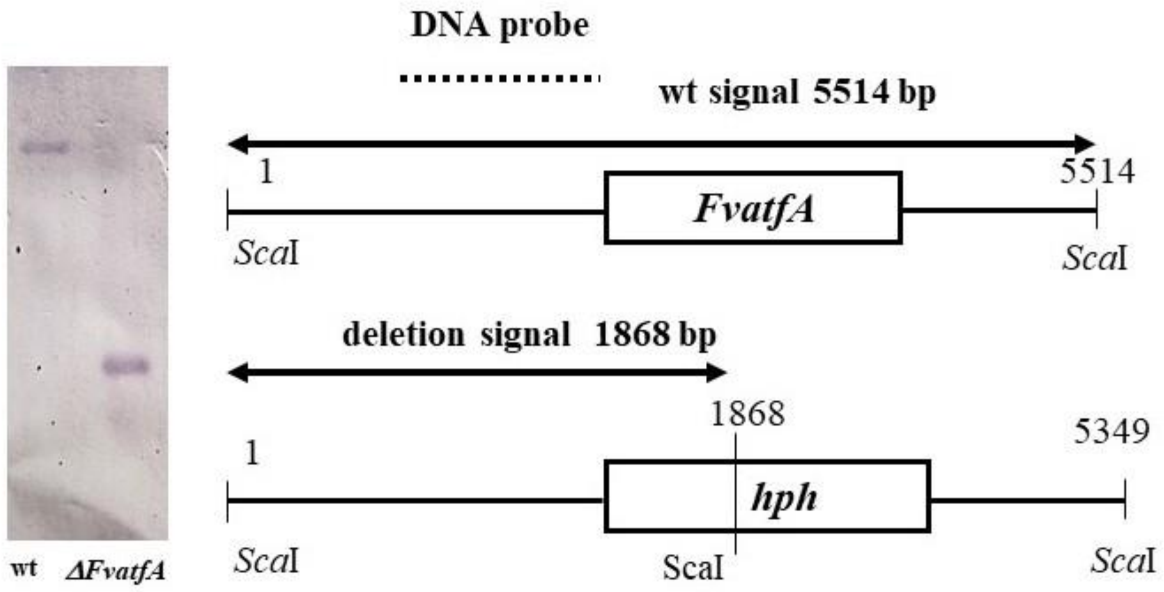

Supplementary Figure S2. Confirmation of the *F. verticillioides atfA* gene deletion on Southern blot. The *Sca*I restriction sites inside an approximately 5.5 kb long genomic region surrounding the *FvatfA* gene are presented in the wild type FGSC 7600 strain and in the *ΔFvatfA* deletion mutant. The upstream noncoding region of the *FvatfA* gene (indicated by a broken line) was amplified by PCR, labelled, and was used as a probe in Southern blotting. The probe hybridized to a 5514 bp restriction fragment in the parental FGSC 7600 strain while in the *ΔFvatfA* mutant it labelled a 1868 bp fragment (see arrows), as expected.

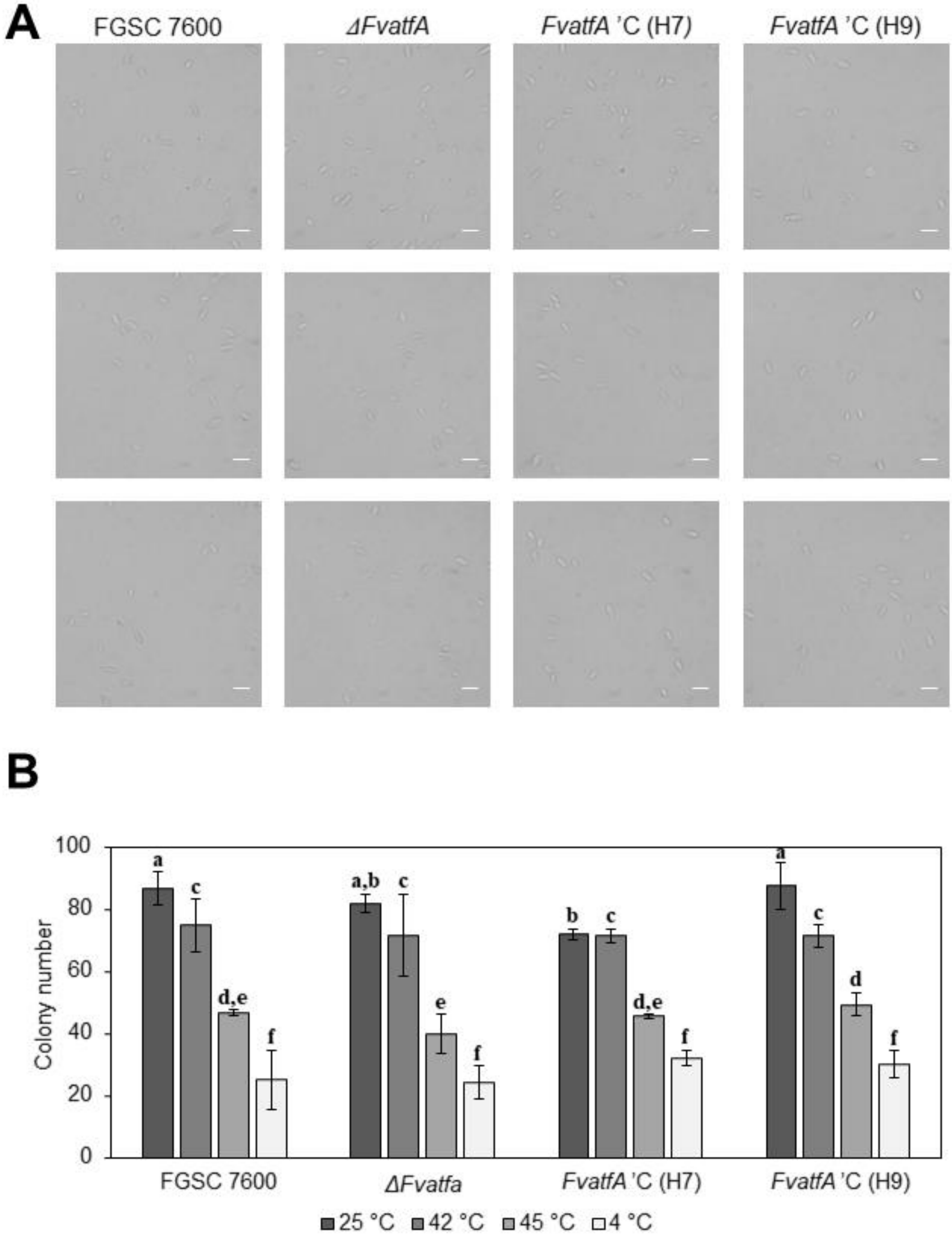

Supplementary Figure S3. Variations in the morphology as well as in the heat and cold tolerances of the conidiospores the *F. verticillioides* FGSC 7600 control, the *ΔFvatfA* mutant and the *ΔFvatfA* 'C H7 and H9 complemented strains. In Part A, a typical set of microscopic photos taken on spore suspensions of the strains are presented. White bars

indicate 10 μm. For statistical evaluation of spore yields and sizes, you can consult Figure 3. As shown in Part B, no significant differences were found in the heat and cold tolerances of the conidiospores. In these experiments, freshly grown conidia were kept at 25, 42 or 45 °C for 60 min or stored at 4 °C for a week. Following that, spore suspensions were spread on Czapek-Dox Agar medium and the colonies were counted after 2 d incubation at 25 °C. Different letters indicate significant differences between the tested strains (one-way ANOVA fallowed by Tukey post-hoc test; adj. $p < 0.05$). For further details of the ANOVA see also Supplementary Table S5.

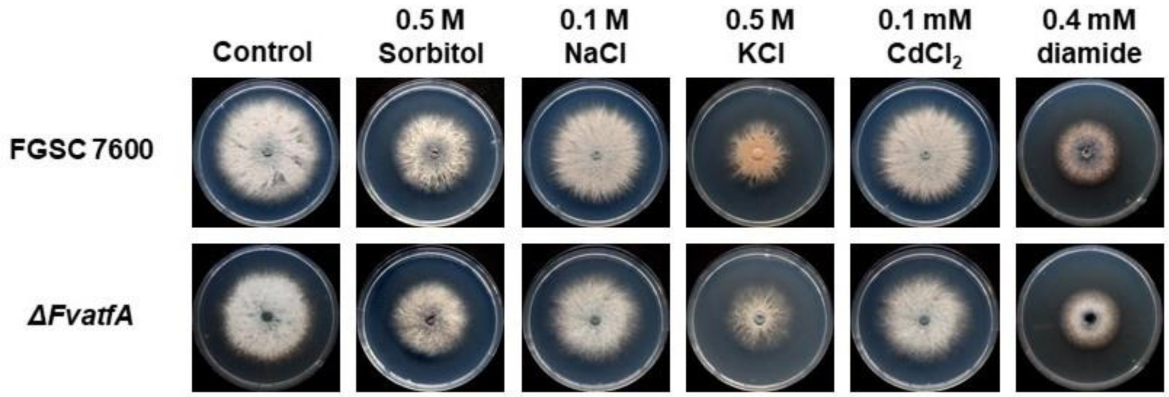

Supplementary Figure S4. Testing and comparing the high-osmolarity, heavy metal and diamide tolerances of the *F. verticillioides* control and *ΔFvatfA* gene deletion mutant strains. A typical set of photos taken on the Czapek-Dox stress agar plates after 6 d incubations at 25 ^{o}C are presented. Please note that all differences observed between the colony diameters of the tested *F. verticillioides* strains in stress agar cultures could be attributed to the slow growth phenotype of the *ΔFvatfA* strain (Figure 1).

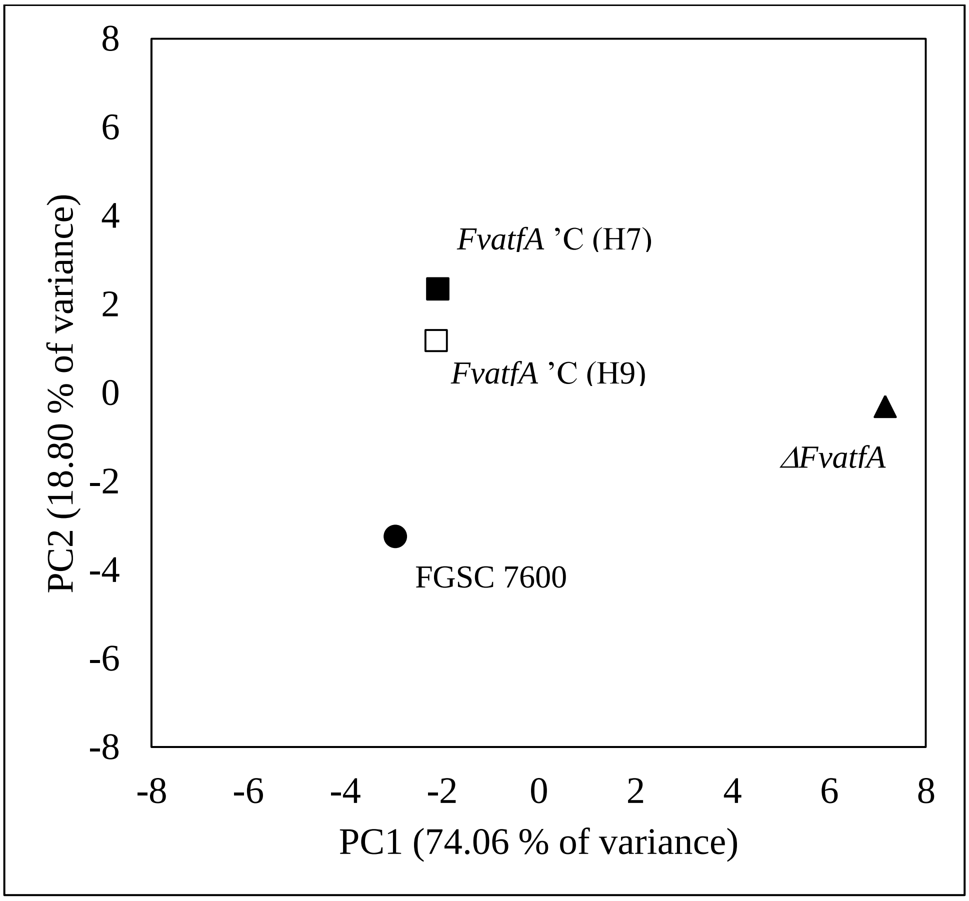

Supplementary Figure S5. Principal component analysis of the studied strains. The separation of the strains in the score plot of PC1 and PC2 can be interpreted as follow: (i) the deletion mutant clearly separated from the other three strains, (ii) the wild-type FGSC 7600 separated from the other three strains, (iii) the complemented strains created a group separating from the wild-type and deletion mutant strains too. PCA was applied on mean values presented in Supplementary Table S5. For more information of the results of the PCA analysis see also Supplementary Table S8.

FVEG_00316 *fum1*

CCACCTCTATGCCAGCCCTGACTCAATGGCACAGCACGCACACCATCACTAACGGTAAACTCAAAACGACAGC
CGCTTGCATTGAGGCTAAAGCACGGTTCCGGCTTATACGAACAGAAGTCTGACATCTTTCATGATAAGAAGAA
GAAGAAGAAGCTGTCGGAATGATACAGACCCAAATCAGATATCCGACCGGCTCCTGGTCCCATTCCATTATGA
GCCAATGGGGCAATTGCCCCCTTTCCCTACCCGAGTGTTTTCGGACGTTACTGTCCCTCACCCGGGAACTCTAG
CAAACTATTGGGATGTAGGATAATAATAGTAACCTTCCTACTGGTTGCCGAATTTCCGGGTCTCTTACTCGTCTT
TGAGGATACTGACAATTGCGCTTCGGAGTATTGCGTGTCCTCTCGGATGGGAGACTAAATCGTATACAACAAC
CGGAATTTCGATGCTGATTGCAATGATAGAGACGAGACAGATATATAACCATGAAATACAGTGTTATTGGAG
ACTCTGTATTTGATCCATCGCAACCTTATTATCGTTTTAAACGCTACTGATATCATCAACGTTCGACATG

FVEG_14635 *fum8*

AATTAACAGTTTTAAAACTTGAAATCAAGATAAACACTTACCAAGCTAGCACTTGACTTGTTGTGTGAAAGTGA
ATTACTCTGTAAACCGAGAAATCCAATATCATGCATTGGCGTTGGCAAAAACAATCCTCTGCTTTTTTGCATGA
ACAATATAACGGAATCTAAATACTCGATGGACCACACGAGTACGTAAAGGTAGCAGCAAATGAAGGCTATAT
AATGAACAAGACGCTCAACTCAATGTATCTCTACTCTCCAAGGAACAGATCTCAAAATGGCTTCCTTCTC
CTGTGCCAAGCTGGGCTGGTACGAGGAATGTTGACTACCTTACCGAATGTGACATCCAGAAAACTGTTG
TATGTACAACTTTCAATTATATGCTACAGGTTGAGCTGAGCTACTAGGTAGGTAGCAATGATTTATCCGT
CACTCACAGAAACAGTGCCCGGAAGCATCTTTTCAAACATCCGACAGATGCCACACGGGGTATCCGAAC
TTCCGAATAACCCCCAGTACAACGTTGGCCTTTTGGATTCTTGGTAGCAGAGTCTACCGATCAGCTACCT
TACCTAGTCAGTCCATCAGTTTCCAGTCGTTGACTCGAAATGAGGAACAGATACAAAATCATCCCGGGTT
ACATTTAAAACTGAGGTTTTCATACAGTGAAAGTCATAACTCTGAACAACTTCATTTTTACCTTGTGTCA
GCCCTTGCATTGCCCATCATG

FVEG_14633 *fum21*

AAAAAATATAGAAGAATAATAGATCAAATATGCCTAAAAGAAATGCTCCCAGACACGATCCTAGGCAAGCAT
AAAAGCTCATATAACTCCATCCACAAGCTCCTCACCAGTGAACGCTGAATACTGTCCTATTAGTCAATTG
GCTCCAGAAACCACGCCCTTTCTTCCAAGTAAAGTGCCCATAACCACTGGATAAGCAAGCAGATGGAAA
ATAGGAATAGACCCTATTACCGAGTAAGTCTTATCGGTAGCTGAGTGTGATGCATCTGGTATATCGTCAA
GTTCAGCCATTATCCATCCAGGTACTCCATATCAAAACGTGTTTCTTCCACTTGCTGGGTTGTATCTTGA
AGTAACAGCGGGACTAGCCTAGGGGTCGGGCCGTCTGCCGTGATAAGCGCTGTGATGTTACGTTATGTC
GACTAAAGTACCGTGCAGGGCATGTTGATGATTGCATGCATTAGTCTACCTAACAGAAACTGCCGTGTT
ATCCGAACAAAACATTGAACTTTGCTGTGTCCTTGGCCATCATGTTAAATCATAAAGCTGCCTTAATTCT
AGGACCCTTCGTAGACCTTGTTTTATATCTTATTCTACTTGTATCATCTGTGATTTATTTCATCATCAACT
TTTGCGATGGCGGGGTCCATTGTGTTTGTGACAGATAATCAGATTAGCGCAAATTCGAGACACAAGCCA
CGATTTAGACGACGTCGTGGCGCGTGTGAGTCGTGTAAGCGTCGTAAAGTCCGGTGTAAGTTGCTTGGC
CTCCTCCACTGCTTTGTACTGTACCTAGCTTATGGTTCTTATGACTACTAGGTAATGGCACAAACCCTTG
CAATCAATGCCAAGTCAGATTCCCGTCAAGATGGTACATAGTCATCTCAGCCCGAGCTAACCAGAAGAG
CAGAAATCGTCGATAGAATGCTTATATAGCAGCTCAAGCTGGAAAGCTAACGACGGAGATACAGAGCGT
GGACCATCAGGATCGCCGGTGCAACATACAAGGGGGAGTTTGACGCCTCCTCAAACGAGCCCATATGGT
GGAATGGCTTGTAATGGATCAGCGGAAGAGTCTTCGAGATTTGATGGCTCGGATGCACACAGCCTTTTG
ATGAGCCTCAGCAATACTGACTGTACGACTAATACTGAGGCAACAGAGATGTCGCAGTGGCAGAATTGG
TTCGTTAGCGACCTACCTCTAGGGAGCGATGTGCAACTTACAGGGACATCTGATGAGTCTTTGACCGAC
TGGTTGGACCCCCAGCTTGTCAACCCAGCAGATACCGCGGCCATTATG

FVEG_10718 *carRA*

GTATGTCAAAGCATACGATACTCCATGAAGCTATACATACAGGATACACCCATCCACAGCCAGCAGACCACCA
GTAACTTAACTATATAGCCAAACCCTCATCAGATCGAGATGATCAGGAAGGACGTAGGTCGCATCAATCG
CTGGATAACACGGCACGCGATGGGAATATCCATCCATCAAAACCACTAGCACAATTTGTCACGCGTCAT
AATCAAGACCCATCTCCATCACGGTAACGGCAATGTCAAAAAGACCGAGTAACCCGAAAAAAAAGGGTC
TCGAGTTAATTGATGACAAGTACACTCGATACCCAAAAGAGTCGAACAAAAAAGTCGTGACGTCAACGA
GTCCCTGAGATGTAACATGACAGAAAACGCCTCTCCCCACTCACACTCAACAATGGTGAGCAGAAGCTA
GCGGTGGGGGAGCATTATTGTCACCCAAGAAATAACGGACAGGGGTAAAGGGCTGTGTTAGTATCTGAA
AAACAAACTGTGGACACAAGCACCACTCACACTCCGACTTCTTCCTCAACAACCTTTTCCTCACAATCTT
TTGACTCTGGCTTTATTTGAAGCCCATATCGTATGCCGTCACGATCTGCTTTTTGTTGTTGTTCACGTAC
ACGAAATAAATAAACCTCTGTCAAAATG

FVEG_10717 *carB*

CATAAACCCACCAACCAATCATTCAACACGCATCACACCGCCAAGTCCACTTAAAGACCCTCCAAACCAAAC
CCGTCAATGATCGTCAACGCATCAACGACTTCAATTCTCCTGTAATCTTCAATGAATCACTAAAATCCCT
CAAAAAACAAACTGCGCCTCGCTAAGCACAGGGTAATCTGGCCTTTCACATGTACGCCAAGTGGTCTCAT
TAGACGAGTGCGAAGGAAAAAGTGGATCAAAACAAAATGGAAGACGTCATTGACTCTATTGCTTTGGCT
ATCAAAACCGTTGGCTTCGGGTGAGTCGGTTACTGACTGAGCATCTTCACTTTTTACAGCAAAAATG

FVEG_09251 *carT*

TAGCTTGATGTTCCGTCAGATGTTTTCATGGAAGCAATTAATGAAACCATGAAAGTAACTATTGTTGCCAAGAC
GATAAAAACCGCCAAGAAAATCGAGTCAGCCATGAAGCAAACATCCAATACCCCGTACCTGAGAGCCAT
CTGACCATTATACATGATTCGCTTGGTTGCTAAGACGAAAGGCTGGTATTCATAATATCTGCATCGGAGT
AACAGTGATACTATAGAATCTTTTGTTCAAGAGAGAATAACTCGTCATGATTATGTTCTCATCACGTTCA
ATAGGGTCAATGTCTTGATTATATCGACCAGGATATGTAATCATCAAGACCAACTTCACTTACCGACATC
GATATCCTCATCGCCAATAAACCACCACCGCCTGATAGATAGTATGTACCACTTCGGAATCTAATAGCCT
ACTCTAAATCCAGCTCAACAAGCAATAAACGCCAGGATCCAGCCAAGAGTCACACACCGGAATCTCGAG
TCGTCAATCCCCCGCCTGCCGTACGCCGCCAAATCCACGATGCTCATGCCAACGAAGCACATTCTCGTC
AAGCCACACTCGCTTTCGCTAACACAGTATCGCAAGTACACGCAAGATGCATCCATCATCCCTTAAACGC
CAACGTCCAAACTAACCATCTTGTCGTCAGCTTTTGATAGGCCCCCCCAAGCGACGAGCGGAACAATAC
CGCACACGCGAAGGCGCACGAAGTATTGAGCCACTAGTGAGTAGGTAGAGTAAATCAAATCGAATCAAC
AACCTCAGCTTTCGGGTGCCGACATGAGTTGTTTTTGTGGTGATTTCTTTCATGCGGAAGTGTCGGGGCT
TGGTATTTTCGTTTTATGGTGCGGAATCCGGTTTGGTTTACTGATGGTAGCGTCATTGACTTTACACAAC
CCATGCGGGAATACAACGAAGCGGGTTTGCGTACTTAATAAGTATGAGATCCATATTTTAAAAATACCCT
ATTATCATGATGCGTTTTGTAAAGAGTTGTGTTTTATTGTTTTAAAGTTGAATGACGACAGTGAAGCTTT
AAAGTGGTTATCTTCACTTGCAGGTTCATCGTCGGTGCCAACACTGCATTGCGTTGCAAACGTACACAGA
CATAGACAACAGGGTGCACTGTGTTGTGATTGTATAGAGCCAATGCTATGTTTCTCACTTGGCACATGAG
CTGAACTTCATTTCACGCTTGTTTTATAGATATATCTTTAGAGCATGCCTCTTGTTCTTGCATCTTTGTTT
CCCAGACCCCAAACGATATTGTCGCAAACGAAACGAAAGCAACTTTTTCTCTCCTATTATTAGCCAATTT
TCGCAAATAAACTTTACTATG

FVEG_03379 *bik1*

ACTAAAGCCAACTGTCAAGGAGAGTCTATTCAAGTGCCGAGATTCGAATCACCATCCAGGGACAGAGCCTCAC
GTAGTTGGTATTCACACATTAGCGCATCGCGTCCGTGGCTCTTCTCTTGACTCGATTCGTCCTCGGTTCACGGCA
CATACTACGCCACCCTGCCCGTTTCAGCTTCCCCTGCAAGATAAGAATTCGGACCTCTTCGGAGCTTCATCT
CTGATCTGATCCTGCGTGGCGTAACTAGCCGCTATACTGACCCTGGCCCACCCATGCAAGCCACTACGG
ACATTTTCGCAAAAGCGCGGAACCAACACTAGCCAGACCCTTACGAATTACCAAAAAGGGTCACGTATG
TTTGAACCTCTACAGTATAAGCCAGATACATCTTGGCGAGAAAATAGCCAAGACAAACGAATATTGCACC
TTTGTTACAGCTTTCAACAGCGATTCTTTTTGCCATCTTACATTTTCAAGATG

Overlapping genes with the tested gene promoters:

Gene	Chain	Overlapping gene	Function	Chain
carRA	-	*carX* (FVEG_10719)	carotenoid oxygenase	+
carB	-	*carRA* (FVEG_10718)	carotene cyclase	-
carT	+	FVEG_09250	NA	-
fum1	+	*fum21* (FVEG_14633)	Zn(II)2Cys6 transcription factor	+
fum8	-	*fum3* (FVEG_00320)	dioxygenase	-
fum21	+	*zbd1* (FVEG_00314)	zinc binding dehydrogenase	+
bik1	-	*bik2* (FVEG_03380)	monooygenase	-

Supplementary Figure S6. Putative ATF/CREB transcription factor binding sites on the promoter regions of the genes involved in secondary metabolite production. Colour code: **ACGT** – 5' untranslated region between TSS and ATG, – first base of the transcription start site (TSS), – poly-A tail (PolA), **ATG** – translation start codon, GTAT – TSS region

of the *carX* gene up-stream of *carRA*, – binding sites between -150 - +50 bp with respect to (w.r.t.) TSS, – binding sites up-stream of position -150 bp w.r.t. TSS, – binding sites down-stream of position +50 bp w.r.t. TSS.

References

Ádám AL, García-Martínez J, Szűcs EP, Avalos J, Hornok L (2011) The *MAT1-2-1* mating-type gene upregulates photo-inducible carotenoid biosynthesis in *Fusarium verticillioides*. FEMS Microbiol Lett 318:76-83. doi: 10.1111/j.1574-6968.2011.02241.x.

Balázs A, Pócsi I, Hamari Z, Leiter É, Emri T, Miskei M, Oláh J, Tóth V, Hegedűs N, Prade RA, Molnár M, Pócsi I (2010) AtfA bZIP-type transcription factor regulates oxidative and osmotic stress responses in *Aspergillus nidulans*. Mol Genet Genomics 283:289-303. doi: 10.1007/s00438-010-0513-z.

Brown DW, Butchko RA, Busman M, Proctor RH (2007) The *Fusarium verticillioides FUM* gene cluster encodes a Zn(II)2Cys6 protein that affects *FUM* gene expression and fumonisin production. Eukaryot Cell 6(7):1210-1218. doi: 10.1128/EC.00400-06

Butchko RA, Brown DW, Busman M, Tudzynski B, Wiemann P (2012) *Lae1* regulates expression of multiple secondary metabolite gene clusters in *Fusarium verticillioides*. Fungal Genet Biol 49(8):602-612. doi: 10.1016/j.fgb.2012.06.003.

Colot HV, Park G, Turner GE, Ringelberg C, Crew CM, Litvinkova L, Weiss RL, Borkovich KA, Dunlap JC (2006) A high-throughput gene knockout procedure for *Neurospora* reveals functions for multiple transcription factors. Proc Natl Acad Sci U.S.A. 103:10352-10357. doi: 10.1073/pnas.0601456103.

Guo M, Guo W, Chen Y, Dong S, Zhang X, Zhang H, Song W, Wang W, Wang Q, Lv R, Zhang Z, Wang Y, Zheng X (2010) The basic leucine zipper transcription factor Moatf1 mediates oxidative stress responses and is necessary for full virulence of the rice blast fungus *Magnaporthe oryzae*. MPMI 23:1053-1068. doi: 10.1094/MPMI-23-8-1053

Hagiwara D, Asano Y, Marui J, Yoshimi A, Mizuno T, Abe K (2009) Transcriptional profiling for *Aspergillus nidulans* HogA MAPK signaling pathway in response to fludioxonil and osmotic stress. Fungal Genet Biol 46(11):868-878. doi: 10.1016/j.fgb.2009.07.003

Hagiwara D, Suzuki S, Kamei K, Gonoi T, Kawamoto S (2014) The role of AtfA and HOG MAPK pathway in stress tolerance in conidia of *Aspergillus fumigatus*. Fungal Genet Biol 73:138-149. doi: 10.1016/j.fgb.2014.10.011

Jiang C, Zhang S, Zhang Q, Tao Y, Wang C, Xu JR (2015) *FgSKN7* and *FgATF1* have overlapping functions in ascosporogenesis, pathogenesis and stress responses in

Fusarium graminearum. Environ Microbiol 17:1245-1260. doi: 10.1111/1462-2920.12561

Johnson ET, Proctor RH, Dunlap CA, Busman M (2018) Reducing production of fumonisin mycotoxins in *Fusarium verticillioides* by RNA interference._Mycotoxin Res 34(1):29-37. doi: 10.1007/s12550-017-0296-8

Lazzaro I, Busman M, Battilani P, Butchko RAE (2012) *FUM* and *BIK* gene expression contribute to describe fumonisin and bikaverin synthesis in *Fusarium verticillioides*. Int J Food Microbiol 160:94-98. doi: 10.1016/j.ijfoodmicro.2012.10.004

Nathues E, Joshi S, Tenberge KB, Driesch M, Oeser B, Bäumer N, Mihlan M, Tudzynski P (2004) CPTF1, a CREB-like transcription factor, is involved in the oxidative stress response in the phytopathogen *Claviceps purpurea* and modulates ROS level in its host *Secale cereale*. MPMI:383-393. doi: 10.1094/MPMI.2004.17.4.383

Nguyen TV, Kröger C, Bönnighausen J, Schäfer W, Jörg B (2013) The ATF/CREB transcription factor Atf1 is essential for full virulence, deoxynivalenol production, and stress tolerance in the cereal pathogen *Fusarium graminearum*. MPMI 26:1378-1394. doi: 10.1094/MPMI-04-13-0125-R

Nimmanee P, Woo PCY, Vanittanakom P, Youngchim S, Vanittanakom N (2014) Functional analysis of atfA gene to stress response in pathogenic thermal dimorphic fungus *Penicillium marneffei*. PLoS One 9(11): e111200. doi:10.1371/journal.pone.0111200

Qi X, Guo L, Yang L, Huang J. (2013) Foatf1, a bZIP transcription factor of *Fusarium oxysporum* f. sp. *cubense* is involved in pathogenesis by regulating the oxidative stress responses of Cavendish banana *(Musa* spp.) Physiol Mol Plant P 84:76-85. doi: 10.1016/j.pmpp.2013.07.007

Sakamoto K, Iwashita K, Yamada O, Kobayashi K, Mizuno A, Akita O, Mikami S, Shimoi H, Gomi K (2009) *Aspergillus oryzae* atfA controls conidial germination and sress tolerance. Fungal Genet Biol 46:887-897. doi: 10.1016/j.fgb.2009.09.004

Takeda T, Toda T, Kominami K, Kohnosu A, Yanagida M, Jones N (1995) *Schizosaccharomyces pombe* atf1+ encodes a transcription factor required for sexual development and entry into stationary phase. EMBO J 14(24):6193-6208. doi: 10.1002/j.1460-2075.1995.tb00310.x

Temme N, Oeser B, Massaroli M, Heller J, Simon A, Collado IG, Viaud M, Tudzynski P (2012) BcAtf1, a global regulator, controls various differentiation processes and

phytotoxin production in *Botrytis cinerea*. Mol Plant Pathol 13:704-718. doi: 10.1111/j.1364-3703.2011.00778.x

Venter JC, Adams MD, Myers EW, Li PW, Mural RJ, Sutton GG, Smith HO, Yandell M, Evans CA, Holt RA, Gocayne JD, Amanatides P, Ballew RM, Huson DH, Wortman JR, Zhang Q, Kodira CD, Zheng XH, Chen L, Skupski M, Subramanian G, Thomas PD, Zhang J, Gabor Miklos GL, Nelson C, Broder S, Clark AG, Nadeau J, McKusick VA, Zinder N, Levine AJ, Roberts RJ, Simon M, Slayman C, Hunkapiller M, Bolanos R, Delcher A, Dew I, Fasulo D, Flanigan M, Florea L, Halpern A, Hannenhalli S, Kravitz S, Levy S, Mobarry C, Reinert K, Remington K, Abu-Threideh J, Beasley E, Biddick K, Bonazzi V, Brandon R, Cargill M, Chandramouliswaran I, Charlab R, Chaturvedi K, Deng Z, Di Francesco V, Dunn P, Eilbeck K, Evangelista C, Gabrielian AE, Gan W, Ge W, Gong F, Gu Z, Guan P, Heiman TJ, Higgins ME, Ji RR, Ke Z, Ketchum KA, Lai Z, Lei Y, Li Z, Li J, Liang Y, Lin X, Lu F, Merkulov GV, Milshina N, Moore HM, Naik AK, Narayan VA, Neelam B, Nusskern D, Rusch DB, Salzberg S, Shao W, Shue B, Sun J, Wang Z, Wang A, Wang X, Wang J, Wei M, Wides R, Xiao C, Yan C, Yao A, Ye J, Zhan M, Zhang W, Zhang H, Zhao Q, Zheng L, Zhong F, Zhong W, Zhu S, Zhao S, Gilbert D, Baumhueter S, Spier G, Carter C, Cravchik A, Woodage T, Ali F, An H, Awe A, Baldwin D, Baden H, Barnstead M, Barrow I, Beeson K, Busam D, Carver A, Center A, Cheng ML, Curry L, Danaher S, Davenport L, Desilets R, Dietz S, Dodson K, Doup L, Ferriera S, Garg N, Gluecksmann A, Hart B, Haynes J, Haynes C, Heiner C, Hladun S, Hostin D, Houck J, Howland T, Ibegwam C, Johnson J, Kalush F, Kline L, Koduru S, Love A, Mann F, May D, McCawley S, McIntosh T, McMullen I, Moy M, Moy L, Murphy B, Nelson K, Pfannkoch C, Pratts E, Puri V, Qureshi H, Reardon M, Rodriguez R, Rogers YH, Romblad D, Ruhfel B, Scott R, Sitter C, Smallwood M, Stewart E, Strong R, Suh E, Thomas R, Tint NN, Tse S, Vech C, Wang G, Wetter J, Williams S, Williams M, Windsor S, Winn-Deen E, Wolfe K, Zaveri J, Zaveri K, Abril JF, Guigó R, Campbell MJ, Sjolander KV, Karlak B, Kejariwal A, Mi H, Lazareva B, Hatton T, Narechania A, Diemer K, Muruganujan A, Guo N, Sato S, Bafna V, Istrail S, Lippert R, Schwartz R, Walenz B, Yooseph S, Allen D, Basu A, Baxendale J, Blick L, Caminha M, Carnes-Stine J, Caulk P, Chiang YH, Coyne M, Dahlke C, Mays A, Dombroski M, Donnelly M, Ely D, Esparham S, Fosler C, Gire H, Glanowski S, Glasser K, Glodek A, Gorokhov M, Graham K, Gropman B, Harris M, Heil J, Henderson S, Hoover J, Jennings D, Jordan C, Jordan J, Kasha J, Kagan L, Kraft C, Levitsky A, Lewis M, Liu X, Lopez J, Ma D, Majoros W, McDaniel J, Murphy S,

Newman M, Nguyen T, Nguyen N, Nodell M, Pan S, Peck J, Peterson M, Rowe W, Sanders R, Scott J, Simpson M, Smith T, Sprague A, Stockwell T, Turner R, Venter E, Wang M, Wen M, Wu D, Wu M, Xia A, Zandieh A, Zhu X. (2001) The sequence of the human genome. Science 291(5507):1304-1351. doi: 10.1126/science.1058040
